# Digital gene expression approach over multiple RNA-Seq data sets to detect neoblast transcriptional changes in *Schmidtea mediterranea*

**DOI:** 10.1186/s12864-015-1533-1

**Published:** 2015-05-08

**Authors:** Gustavo Rodríguez-Esteban, Alejandro González-Sastre, José Ignacio Rojo-Laguna, Emili Saló, Josep F Abril

**Affiliations:** Departament de Genètica, Facultat de Biologia, Universitat de Barcelona (UB), and Institut de Biomedicina de la Universitat de Barcelona (IBUB), Av. Diagonal 643, Barcelona, 08028 Catalonia Spain

**Keywords:** Planaria, Neoblast, Stem cell, Transcriptome, Transcription factor

## Abstract

**Background:**

The freshwater planarian *Schmidtea mediterranea* is recognised as a valuable model for research into adult stem cells and regeneration.

With the advent of the high-throughput sequencing technologies, it has become feasible to undertake detailed transcriptional analysis of its unique stem cell population, the neoblasts. Nonetheless, a reliable reference for this type of studies is still lacking.

**Results:**

Taking advantage of digital gene expression (DGE) sequencing technology we compare all the available transcriptomes for *S. mediterranea* and improve their annotation. These results are accessible via web for the community of researchers.

Using the quantitative nature of DGE, we describe the transcriptional profile of neoblasts and present 42 new neoblast genes, including several cancer-related genes and transcription factors. Furthermore, we describe in detail the *Smed-meis-like* gene and the three Nuclear Factor Y subunits *Smed-nf-YA*, *Smed-nf-YB-2* and *Smed-nf-YC*.

**Conclusions:**

DGE is a valuable tool for gene discovery, quantification and annotation. The application of DGE in *S. mediterranea* confirms the planarian stem cells or neoblasts as a complex population of pluripotent and multipotent cells regulated by a mixture of transcription factors and cancer-related genes.

**Electronic supplementary material:**

The online version of this article (doi:10.1186/s12864-015-1533-1) contains supplementary material, which is available to authorized users.

## Background

During the last decade, there has been increasing interest in the use of *Schmidtea mediterranea* as a model organism for the study of stem cells. These freshwater planarians contain a population of adult stem cells known as neoblasts, which are essential for normal cell renewal during homeostasis and which confers them with amazing regeneration capabilities [1-4]. Although a number of studies based on massive RNA interference (RNAi) [5], gene inhibition [6], microarray [7], and proteomics [8,9] approaches have been carried out to identify the crucial neoblast genes responsible for their stemness, our understanding of their biology is far from complete. The use of next generation sequencing (NGS) technologies provides an opportunity to study these cells in depth at a transcriptional level. For that to be accomplished, however, a reliable transcriptome and genome references are required. Up to eight versions of the transcriptome for this organism have been published to date, making use of different RNA-Seq technologies [10-16], including one meta-assembly which slightly improves each one separately [17]. Despite all these efforts, a consistent reference transcriptome is still lacking.

Some studies have provided quantitative data on transcripts and their respective assemblies, focusing on regeneration [13,17,18] or directly on neoblasts [11,14,15,19]. However, RNA-Seq suffers from an intrinsic bias that affects the quantification of transcript expression in a length-dependent manner. This bias is independent of the sequencing platform and cannot be avoided nor removed by increasing the sequencing coverage or the length of the reads. Furthermore, it cannot be corrected a posteriori during the statistical analysis (by transcript length normalization, for instance). Consequently, the quantification of the transcripts and the detection of differentially expressed genes is compromised [20-22]. Digital gene expression (DGE) [23] is a sequence-based approach for gene expression analyses, that generates a digital output at an unparalleled level of sensitivity [22,24]. The output is highly correlated with qPCR [25-27] and does not suffer from sequence-length bias. The combination of DGE and RNA-Seq data has been shown to help overcome the specific limitations of RNA-Seq [28], and the usefulness of DGE has been thoroughly demonstrated in research ranging from humans [26,29] to non-model organisms [22,24]. However, to date, DGE has not been extensively applied to the study of the planarian transcriptome.

Here, we have compiled and analyzed all the transcriptomic and genomic data available for *S. mediterranea* using DGE. This has facilitated an improved annotation and provided tools to ease the comparison and browsing of all the information available for the planarian community.

We have taken advantage of the resolution of DGE to quantitatively characterize isolated populations of proliferating neoblasts, their progeny, and differentiated cells through fluorescence-activated cell sorting (FACS) [30,31]. The resulting changes in transcription levels were analyzed to obtain transcript candidates for which an extensive experimental validation was performed. This has yielded new neoblast-specific genes, including many transcription factors and cancer-related homologous genes, confirming the validity of our strategy and the utility of the tools that we have implemented. Moreover, we provide a deeper molecular description of four of those candidates, the *Smed-meis-like*, and the three subunits of the Nuclear Factor Y (NF-Y) complex *Smed-nf-YA*, *Smed-nf-YB-2*, and *Smed-nf-Y-C*. Both families of genes are attractive candidates to be studied in planaria. The Meis family of transcription factors specify anterior cell fate and axial patterning [32], whereas the NF-Y complex is a heterotrimeric transcription factor that promotes chromatin opening and is involved in the regulation of a wide number of early developmental genes [33].

## Results and discussion

**Figure 1:**
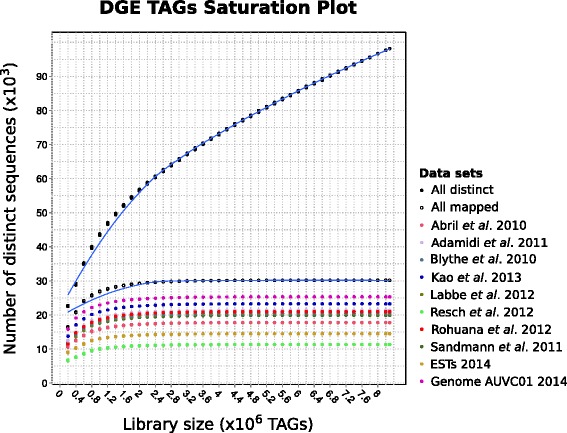
Saturation plot for the distinct tags mapped over each reference data set. Tag sequences were randomly taken to build, by steps of 200,000 tags, increasing-size libraries that were then mapped against the reference data sets. Saturation is reached for libraries around two million tags.

Three DGE libraries were obtained from FACS-isolated cell populations X1 (proliferating stem cells, S/G2/M), X2 (a mix of stem cell progeny and proliferating, G0/G1), and Xin (differentiated cells, G0/G1) [30] (Additional file 1). 8,298,210 total reads were sequenced (X1: 3,641,099; X2: 3,488,712; Xin: 1,168,399), representing 98,156 distinct tags (X1: 70,849; X2: 24,621; Xin: 25,221), with an average of 84.5 reads per tag (X1: 51.4; X2: 141.7; Xin: 46.3). The distribution of the tags in each cell population can be observed in Additional file 2A. DGE is reported to achieve near saturation in genes detected after 6-8 million tags [22]. Furthermore, for moderately to very highly expressed genes (>2 cpm) it occurs with three or even just two million tags [22,34]. Figure 1 shows that saturation was reached at around two million tags for most of the data sets which the distinct tags were mapped to, although the slope for the total number of distinct tags decreases without saturating. It is worth noting that all the reference transcriptome sets performed similarly, achieving a maximum near 20,000 mapped tags. However, when looking at how many distinct tags map to any of those transcriptomes, about 5,000 tags appear not to be shared among all of them (see the “All mapped” and the “All distinct” data series on Figure 1, and further details on mapping below).

A critical point in this kind of experiment has to do with the number of times a tag has to be seen so that it can be considered reliable. Discarding too many tags in an attempt to increase reliability will result in a loss of information whereas keeping all of them may generate background noise. To estimate the specificity of our tags and to establish an optimal cutoff for the minimum number of counts a tag should have in order not to be considered artefactual, we performed a series of simulations mapping iteratively randomized sets of our data. The results are summarized in Additional file 3 for the different cutoffs tested (1, 5, 10, 15 and 20 minimum occurrences of tags). For cutoffs higher than five there is no substantial gain in terms of specificity (the number of hits decreases less than one order of magnitude). Thus, we defined reliable tags as those sequenced five times or more and discarded the rest. Thereafter, for the subsequent computational and experimental analyses, only those tags occurring at least five times were considered. From the initial set of 98,156 distinct tags, 40,670 passed that cutoff (Additional file 2B).

The low technical variability of DGE and its high reproducibility, together with the digital quantification of transcripts, enables direct comparison of samples across different experiments, even from different laboratories [21,22,24-26,29,35]. That property allowed us to contrast our results with those from Galloni [36], who used DGE to identify neoblast genes by comparing irradiated versus control animals over the same strain of clonal *S. mediterranea*. A Venn diagram showing the similarity of the strategies can be seen in Additional file 4. From the total distinct tags, 31.38% (30,806 out of 98,156) were sequenced 10 times or more in our study, compared with just 11,28% (42,159 out of 373,532) in the irradiation strategy, indicating a greater representation of each tag. This suggests, as expected, that the cell-sorting approach has higher specificity. In addition, the strand-specific nature of DGE allows the discrimination of sense and antisense transcripts. Almost 30% of the transcripts successfully identified also presented antisense transcription, even though at lower levels than canonical transcription. This confirms the findings of the aforementioned study in planarians [36] and others [37], and shows that a large proportion of the genome is transcribed from both strands of the DNA. Although the purpose of these transcripts is still open to debate, evidences point to a post-transcriptional gene regulatory function [38].

### Tag mapping to reference sequence data sets

An essential step in DGE is the recovery of the transcript represented by each tag. The nature of the DGE methodology, which generates reads of only 21 nucleotides, implies mapping short reads against a reference genome or a collection of ESTs to retrieve full-length sequences for the original transcripts. On the other hand, the short length facilitates the fast mapping of the tags against the reference sequence data set. To obtain the maximum number of transcripts, tags were mapped against the 94,876 *S. mediterranea* ESTs from the NCBI dbEST[39-42] and all the available transcriptomes (formally those can also be considered as ESTs libraries). 26,822 tags (65.95%) mapped over at least one set of ESTs/transcripts, leaving a huge number (34.05%) unmapped.

**Figure 2:**
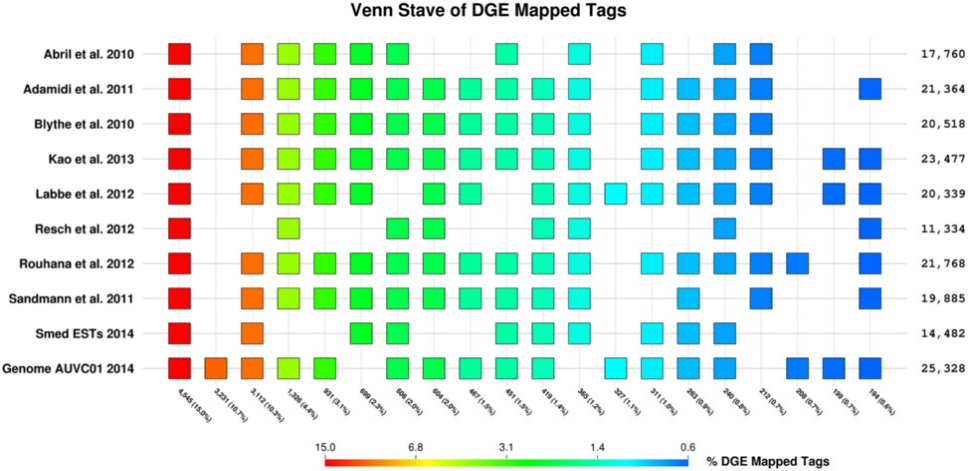
Venn stave showing the proportions of the distinct tags mapped over the different reference data sets. Integrating data for Venn diagrams for sets larger than four or five can be a challenging task, so that, a linear projection of such a diagram is provided in the stave—showing the 20 topmost scoring comparisons from 752 different subsets, accounting for 62.26% (18,710 out of 30,053) of total mappings—for ten reference sequence sets: eight transcriptomes, the *S. mediterranea* ESTs from NCBI dbESTs [39-42], and the latest genome draft AUVC01 [43,44]. Color gradient scale is provided on the bottom bar and it is proportional to the number of unique tags mapped over each sequence subset. X-axis ticks present the number of tags and their relative percent; the numbers on the right Y-axis correspond to the total number of tags mapped into a given sequence sets comparison. It is easy to spot that 15% of the unique reads are mapping onto all the sequence sets.

In an attempt to recover tags that did not map over the transcripts, tags were also mapped over the *S. mediterranea* genome assembly draft AUVC01 masked with the *S. mediterranea* repeats [23,43-45] (Table 1 and Figure 2). The overlap between transcriptomes was high. Although in most cases sets of reads mapping over a single transcriptome has a very low incidence, there were two cases where one could find a relatively small number of tags mapping to only one transcriptome: 327 tags (1.1%) for Labbé et al. 2012; 208 tags (0.7%) for Rohuana et al. 2012; 3,231 tags (10.7%) remarkably mapping only over the genome; and 26.1% of tags (10,617 out of 40,670) not mapping at all. For tags sequenced 10 times or more, the proportion of unmapped tags is similar: 20.5% (6,327 out of 30,806) (Additional file 2B). Even allowing up to two mismatches, 9.36% of the reads remain not mappable to the genome. This is still an important amount, considering that two mismatches is very permissive (it represents almost a 10% of nucleotide substitution in the read with respect to the reference sequence).

**Table 1:** **Summary of mapped tags**

**Reference**	**Mapped**	**One match**	**More than one match**	**Orphan**	**Contigs per tag**
Abril et al. 2010	17,760	12,848	4,912	22,910	1.616
Adamidi et al. 2011	21,364	18,024	3,340	19,306	1.282
Blythe et al. 2010	20,518	17,649	2,869	20,152	1.204
Kao et al. 2013	23,477	15,791	7,686	17,193	1.444
Labbé et al. 2012	20,339	19,513	826	20,331	1.040
Resch et al. 2012	11,334	9,789	1,545	29,336	1.158
Rouhana et al. 2012	21,768	14,891	6,877	18,902	1.579
Sandmann et al. 2011	19,885	14,774	5,111	20,785	1.407
ESTs 2014	14,482	3,650	10,832	26,188	5.442
Genome AUVC01 2014	25,328	19,019	6,309	15,342	1.272

Counts for the tags mapping over the reference data sets depicted in Figure 2. Total (distinct) tags: 40,670; mapped tags: 30,053; orphan tags (tags not mapped): 10,617.

These results indicate that there will be a significant number of transcripts that are not represented yet neither in the current transcriptomic sets nor in the reference genome, despite their coverage depth [46-49], and may correspond, for instance, to weakly expressed genes [50]. Mapping tags are expressed on average at 50.78 cpm, while non-mapping tags only at 19.85 cpm. Nonetheless, since transcriptomes currently available lack the complete annotation of 3’-UTR regions and the DGE libraries were made from the 3’-ends, reads that map to genomic sequences but not to current transcripts may potentially come from the 3’-UTR ends not yet sequenced. To evaluate this possibility, we have projected the transcriptome from Kao et al. 2013 [17] over the genome and looked for the proximity of the tags mapping next to the 3’-end of the transcripts (Additional file 5). Downstream sequenced DGE tags account for 4.12% of all the possible CATG targets. This small amount of sequenced tags only mapping to the genome may correspond to potential novel unsequenced transcripts, alternative 3’-UTR exons of splicing isoforms, misannotated or alternative poly-adenylation sites, or even to non-coding RNAs not represented yet in the present transcriptome sets. Future RNA-Seq experiments may provide further sequence evidences supporting transcripts for those tags.

### Functional annotation

**Figure 3:**
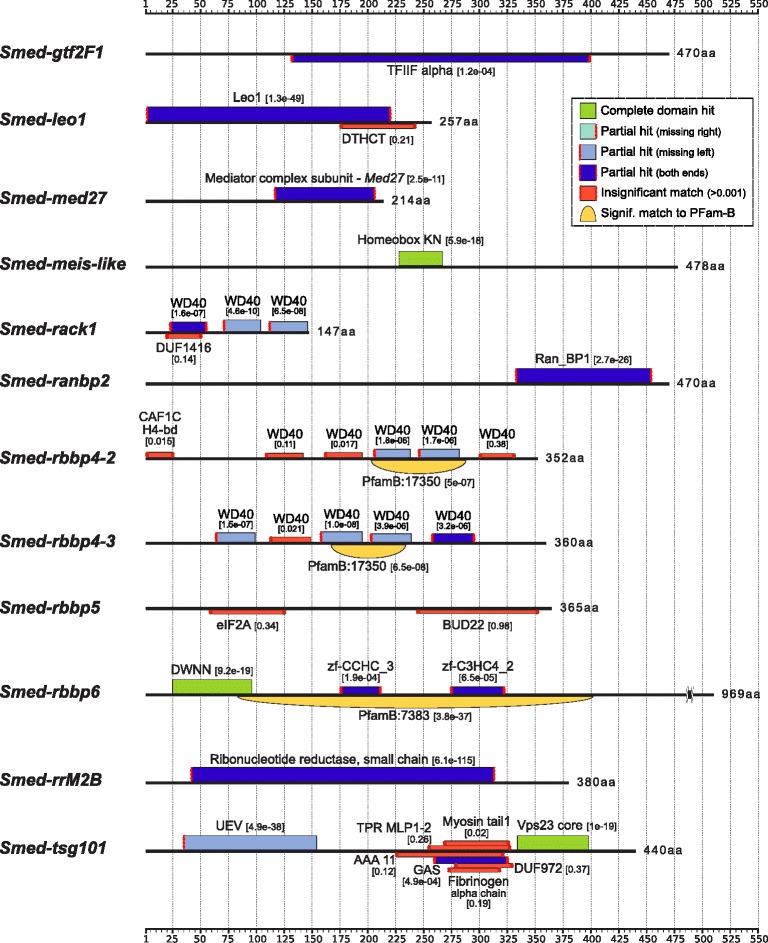
Predicted functional domains for several of the selected transcript candidates. Functional domains annotation based on Pfam hidden Markov models. Legend box shows a classification of the domain hits based on its match to complete domain model; the boxes height is proportional to the E-value score provided for each match. Significant matches were considered for HMMER [117] E-value < 0.001; however, low-significance matches are also shown, as well as hits to Pfam-B models produced by automated alignment protocols. Further annotation over Smed454 transcripts is already available at the GBrowse2 URL planarian.bio.ub.edu/gbrowse/smed454_transcriptome; an example can also be found on Figure 4.

8,903 contigs from Smed454_90e—Smed454 from now on—[10] showing significant expression changes (*p* < 0.001) were selected and, from those, 7,735 contigs presented a hit to a Pfam domain model (Figure 3). For those sequences having a significant hit to a known domain/protein, gene ontology (GO) analysis was performed in order to summarize changes on the biological processes and molecular functions due to the observed expression patterns of the enriched sets of transcripts. Those transcripts were classified according to the cell type in which they were mostly expressed, then their significant GO annotations were clustered (also taking into account their parent nodes in the ontology), to calculate the terms abundance log-odds ratio. Comparison of GO categories between transcripts predominantly expressed in X1, X2 or Xin cell fractions revealed significant patterns of enrichment as indicated in Additional file 6 (see also the “Transcriptomes” tables available from the web site—planarian.bio.ub.edu/SmedDGE—for specific GO terms assigned to each transcript).

The GO comparison between the neoblast population (X1) and the differentiated cells (Xin) reflects distinct functional signatures: X1 is enriched in ubiquitin-dependent protein catabolic process, nucleic acid binding, RNA-binding, helicase activity, ATP binding, translation, and nucleosome assembly; Xin most represented categories include actin binding, actin cytoskeleton organization, small GTPase mediated signal transduction, proteolysis, and calcium ion binding; whereas in X2, markers of secretory activity such as vacuolar transport are more abundant.

### Browsing data

**Figure 4:**
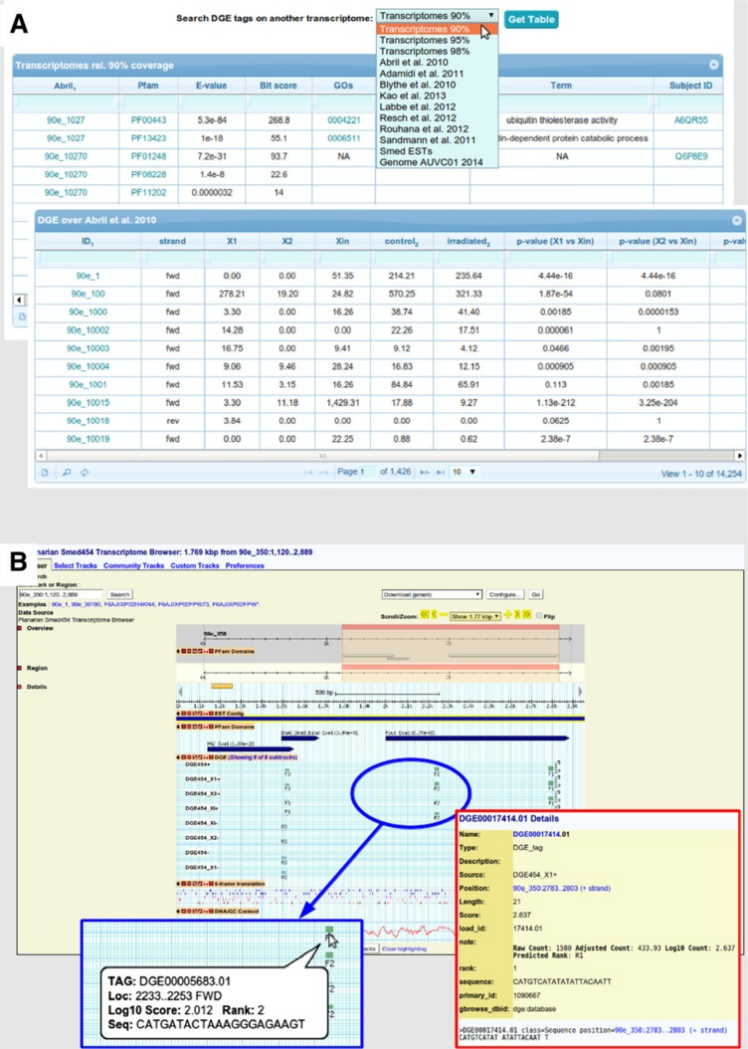
Online data sets and DGE data on Smed454 GBrowse2.**A** - To facilitate browsing of mapped tags over the transcripts we have worked with, we provide a dynamic table interface that paginates through the huge lists of records. This jQuery [112] interface allows the user to easily sort the output table by a given column—just by clicking on the column label—or to search for specific values on the cells—using either the form box just below the column labels or the advanced search available from the magnifying glass icon at the bottom of the table. Three tables, like the one in the background, contain the equivalences between contigs from different transcriptomes, as well as functional annotations, always focusing on the Smed454 data set. The other tables, like the one in the foreground, contain the tag mappings for each single transcriptomes considered to date. **B** - Previously published Smed454 database [10] has been ported to GBrowse2 in order to facilitate navigating through the transcripts annotations, such as predicted domains from Pfam, assembly reads mapping, etc. This panel shows the annotations on *Smed-wi-3* homologous contig as an example. A customized track allows the integration of information about mapped DGE tags into single or combined tracks; tags are represented as boxes with height proportional to log of the normalized tag counts, the rank and the strand for the tag hit are shown in the label just below that box. Bottom left blue box zooms into one of those combined tracks to visualize the pop-up box that the user can recover when moving the mouse over a given tag feature. In addition, bottom right red box displays the details page one can get when clicking on a tag feature.

All tag mappings over the different transcriptome versions are available in the form of dynamic tables from our web site (planarian.bio.ub.edu/SmedDGE, Figure 4A). The relationship between Smed454, along with their domains and functional annotation, with the other reference transcriptomes described in this manuscript can be browsed on a subset of those tables. In order to establish the correspondence between the transcriptomes, a megablast—NCBI BLAST+ 2.2.29 [51]—was performed, filtering the resulting hits afterwards by three levels of coverage (90%, 95% and 98%). Although the focus is set on Smed454, the user can reorder those tables by columns containing identifiers for other transcriptome versions or she can choose to jump to the transcriptome version specific summary table.

Moreover, the Smed454 contig browser [10,52]has been revamped into a more flexible interface based on GBrowse2 (planarian.bio.ub.edu/gbrowse/smed454_transcriptome). One can find there different types of annotation tracks: reads coverage, homology to known genes/proteins, hits to Pfam domains, and also the information of the tags mapped over the sequence. One track-specific GBrowse2 Perl module was modified to display DGE tags data, such as the sequence, counts and rank position. Further customization of the GBrowse2 configuration facilitates the access to most of that information in the form of pop-up summary boxes, but also by means of additional “Details” page (see yellow panel on the right side of Figure 4B).

This browser has been developed under the principle of easy accessibility, in the hope that it will become a useful and informative user friendly tool for experimental researchers in their daily work.

### Experimental validation

**Figure 5:**
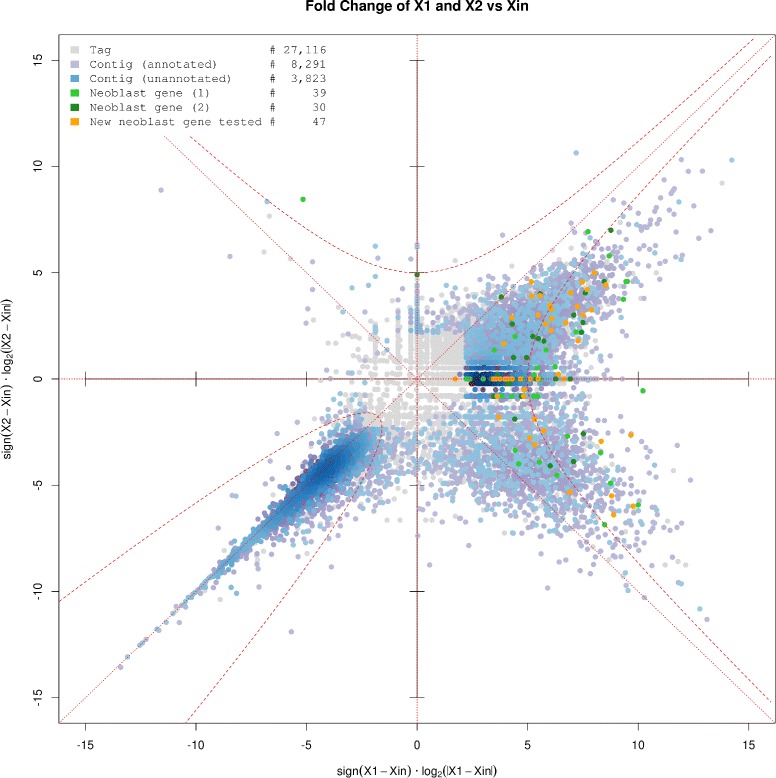
Splashplot projection of the X1/X2 versus Xin expression changes. X-axis represents tags fold change of X1 with respect to Xin, while Y-axis corresponds to fold change differences between X2 and Xin. Fold change is here calculated as the log base 2 of absolute value of difference between X1, or X2, and Xin, while the direction of the change will be given by the sign of that subtraction. Each of the figure quadrants provide insights on tags expression considering the three cell fractions simultaneously. Upper right quadrant contain tags being overexpressed in both X1 and X2 with respect to Xin; bottom left quadrant has those tags overexpressed in Xin versus the other two fractions. Points over the X-axis or Y-axis correspond to tags for which expression levels change only in one cell fraction, X1 or X2, with respect to Xin. The shift trend on most points towards the right vertical hyperbola reflects a higher expression level in X1 when compared to X2 or Xin (otherwise points will fit closer to both diagonals).

The validity of our approach is corroborated by the expression levels detected in 40 already known and well-characterized neoblast genes (Table 2), plus another 29 genes described in the literature with evidence of also being neoblast related (Table 3). As can be observed in Figure 5, both sets of genes show the expected expression pattern along the vertical right hyperbola, indicating a clear X1 specificity, with two exceptions overrepresented in X2: *Smed-nlk-1* and *Smed-prog-1*, which is described to be found in postmitotic cells [53]. *Smed-dlx* and *Smed-sp6-9* are key genes in eye formation [54]; despite their localized activation, DGE was sensitive enough to identify both of them predominantly in the X1 subfraction. Moreover, we could detect expression of genes such as *Smed-smg-1*—which is described as broadly expressed through all tissues, including neoblasts [55]—in both neoblasts and differentiated cells. Finally, 133 clones from two different studies [6,56] focussing on regeneration, stemness and tissue homeostasis are, indeed, significantly overexpressed in neoblasts (Additional file 7).

**Table 2:** **Neoblast genes**

**Gene**	**X1**	**X2**	**Xin**	**p-val X1-Xin**	**p-val X2-Xin**	**Accession**	**PubMed**
*Smed-bruli*	212.57	122.68	0	2.20e-062	1.58e-035	DQ344977	16890156
*Smed-chd4*	159.84	18.34	13.69	5.91e-032	1.10e-001	GU980571	20223763
*Smed-coe*	10.16	0	0	9.77e-004	1	KF487109	25356635
*Smed-cycD*	18.95	0	0	1.91e-006	1	JX967267	23123964
*Smed-dlx*	5.22	0	0	3.12e-002	1	JN983829	21852957
*Smed-e2f4-1*	141.72	23.50	29.96	6.24e-018	7.79e-002	JX967265	23123964
*Smed-egfr-3*	19.50	0.57	0	1.91e-006	5.00e-001	HM777016	21458439
*Smed-egr-1*	510.01	37.26	153.20	9.16e-045	3.06e-017	JF914965	21846378
*Smed-foxA*	15.65	0	0	1.53e-005	1	JX010556	24737865
*Smed-hdac-1*	1086.49	0	60.77	4.19e-122	4.34e-019	JX967266	23123964
*Smed-hnf4*	30.21	8.31	8.56	3.85e-004	1.85e-001	JF802199	21566185
*Smed-hsp60*	113.43	10.32	33.38	8.64e-011	2.18e-004	GU591874	21356107
*Smed-hsp70*	326.28	0	11.13	3.98e-081	4.88e-004	GU591875	21356107
*Smed-jnk*	87.61	13.47	11.98	8.29e-016	1.55e-001	KC879720	24922054
*Smed-lst8*	43.12	1.43	0	1.14e-013	5.00e-001	JN815261	22479207
*Smed-msh2*	57.13	2.58	0	6.94e-018	1.25e-001	JF511467	21747960
*Smed-nanos*	39.27	1.15	0	1.82e-012	5.00e-001	EF153633	17390146
*Smed-ncoa5*	48.34	30.38	0	1.46e-011	5.96e-008	KF668097	24268775
*Smed-nf-YB*	11.26	2.58	0	4.88e-004	1.25e-001	HM100653	20844018
*Smed-p53*	5.22	5.73	0	3.12e-002	1.56e-002	AY068713	12421706
*Smed-papbc*	46.96	0	0	7.11e-015	1	HM100651	20844018
*Smed-pbx*	226.03	38.12	19.69	1.17e-044	6.41e-003	KC353351	23318635
*Smed-pcna*	728.63	24.08	0	3.51e-217	5.96e-008	EU856391	18786419
*Smed-prmt5*	43.67	0.57	0	5.68e-014	5.00e-001	JQ035529	22318224
*Smed-prog-1*	1.92	389.54	37.66	7.09e-010	7.42e-074	JX122762	18786419
*Smed-runt-1*	16.48	0	0	1.53e-005	1	JF720854	21846378
*Smed-sd-1*	14.28	0.57	0	6.10e-005	5.00e-001	KF990481	24523458
*Smed-sd-2*	4.67	0	0	3.12e-002	1	KF990482	24523458
*Smed-smB*	461.12	0	29.96	1.72e-099	9.31e-010	GU562964	20215344
*Smed-smg-1*	72.78	11.47	26.53	1.51e-006	4.38e-003	JF894292	22479207
*Smed-soxP-1*	15.11	3.15	0	3.05e-005	1.25e-001	JQ425151	22385657
*Smed-sp6-9*	38.72	0.57	0	1.82e-012	5.00e-001	JN983830	21852957
*Smed-srf*	40.37	0.29	16.26	5.78e-004	1.53e-005	JX010474	22549959
*Smed-tert*	19.22	0	0	1.91e-006	1	JF693290	22371573
*Smed-tor*	31.86	0	10.27	3.35e-004	9.77e-004	JF894291	22479207
*Smed-vasa-1*	1209.52	22.93	22.25	3.39e-162	1.17e-001	JQ425140	22385657
*Smed-wi-1*	644.59	13.47	0	6.01e-192	1.22e-004	DQ186985	16311336
*Smed-wi-2*	724.78	50.45	26.53	1.41e-176	2.90e-003	DQ186986	16311336
*Smed-wi-3*	433.93	76.82	21.40	9.76e-101	4.01e-009	EU586258	18456843
*Smed-xin-11*	26.64	0	0	7.45e-009	1	DQ851133	17670787

X1, X2 and Xin DGE expression levels of already known and deeply characterized neoblast genes.

**Table 3:** **Likely neoblast genes**

**Gene**	**X1**	**X2**	**Xin**	**p-val X1-Xin**	**p-val X2-Xin**	**Accession**	**PubMed**
*Smed-armc1*	20.60	2.01	0	4.77e-007	2.50e-001	JQ425158	22385657
*Smed-ash2*	17.58	0.86	0	3.81e-006	5.00e-001	KC262336	23235145
*Smed-cpsf3*	19.77	0	0	9.54e-007	1	KJ573358	24737865
*Smed-da*	13.46	0	0	1.22e-004	1	KF487093	24173799
*Smed-eed-1*	42.02	0	0	2.27e-013	1	JQ425136	22385657
*Smed-ezh*	31.03	2.01	0	4.66e-010	2.50e-001	JQ425137	22385657
*Smed-fer3l-1*	12.36	1.15	0	2.44e-004	5.00e-001	KF487094	24173799
*Smed-fhl-1*	158.19	8.31	23.11	2.79e-025	3.67e-003	JQ425148	22385657
*Smed-hcf1*	20.60	0	0	4.77e-007	1	KC262343	23235145
*Smed-hesl-3*	26.09	0.57	0	1.49e-008	5.00e-001	KF487112	24173799
*Smed-junl-1*	173.30	4.59	0	1.97e-050	3.12e-002	JQ425155	22385657
*Smed-khd-1*	29.94	4.87	8.56	3.85e-004	1.22e-001	JQ425142	22385657
*Smed-mcm7*	351.82	24.08	0	5.22e-104	5.96e-008	KJ573361	24737865
*Smed-mll5-2*	97.50	15.48	32.52	7.09e-008	3.88e-003	KC262344	23235145
*Smed-mrg-1*	53.28	3.44	0	1.11e-016	1.25e-001	JQ425133	22385657
*Smed-nlk-1*	0	30.10	0	1	9.31e-010	JQ425157	22385657
*Smed-nsd-1*	135.40	8.03	0	4.23e-039	3.91e-003	JQ425134	22385657
*Smed-pabp2*	191.98	2.58	8.56	2.84e-045	5.37e-002	KJ573359	24737865
*Smed-rbbp4-1*	121.67	0	0	3.13e-035	1	JQ425135	22385657
*Smed-sae2*	19.77	6.02	0	9.54e-007	1.56e-002	KJ573350	24737865
*Smed-setd8-1*	10.99	0.57	0	4.88e-004	5.00e-001	JQ425139	22385657
*Smed-soxP-2*	37.63	4.01	0	3.64e-012	6.25e-002	JQ425152	22385657
*Smed-soxP-3*	14.01	14.62	0	6.10e-005	3.05e-005	JQ425153	22385657
*Smed-sz12-1*	76.90	0	0	6.62e-024	1	JQ425138	22385657
*Smed-tcf15*	47.51	16.05	0	3.55e-015	1.53e-005	JQ425150	22385657
*Smed-vasa-2*	491.06	184.02	55.63	6.25e-087	2.39e-016	JQ425141	22385657
*Smed-wdr82-2*	195.55	16.63	0	2.66e-057	7.63e-006	KC262342	23235145
*Smed-zmym-1*	180.99	6.31	0	8.05e-053	1.56e-002	JQ425146	22385657
*Smed-znf207-1*	44.77	3.73	0	2.84e-014	6.25e-002	JQ425147	22385657

X1, X2 and Xin DGE expression levels of genes described in the literature with some evidences of being neoblast genes.

Based on their X1/Xin expression ratio, we selected a collection of potential new neoblast genes among the most represented in the X1 population. With the chosen candidates we performed expression pattern analysis by whole mount in situ hybridization (WISH) in irradiated animals. At different times after irradiation, as the neoblasts and its progeny decline, the hybridizationsignal disappears [57]. The expression of 42 out of 47 genes tested was diminished or completely lost in irradiated animals (Table 4 and Additionalfile 8).

**Table 4:** **New neoblast genes experimentally validated**

**Gene**	**X1**	**X2**	**Xin**	**p-val X1-Xin**	**p-val X2-Xin**	**TR**	**TF**	**ED**	**CC**	**OG**	**Accession**	**PubMed**
*Smed-atf6A* (*Smed-atfl1*)	12.36	0.57	0	2.44e-004	5.00e-001	∙	∙				JX010554	22549959
*Smed-ccar1*	184.01	79.11	0	1.02e-053	1.65e-024	∙		∙	∙	∙	KM981922	
*Smed-dnaJA3*	133.75	26.94	10.27	2.65e-028	2.53e-003	∙				∙	KM981923	
*Smed-ergic3*	66.74	4.87	0	6.78e-021	3.12e-002					∙	KM981924	
*Smed-got2 ∥ Smed-maspat*	106.29	7.17	0.86	1.78e-030	3.12e-002					∙	KM981925	
*Smed-gtf2E1*	36.25	14.91	0	1.46e-011	3.05e-005	∙	∙				KM981926	
*Smed-gtf2F1*	25.54	0	0	1.49e-008	1	∙	∙				KM981927	
*Smed-hadhB*	153.80	12.33	1.71	4.86e-043	5.55e-003					∙	KM981928	
*Smed-hnrnpA1/A2B1*	341.93	13.76	21.40	3.70e-075	6.75e-002			∙			KM981929	
*Smed-leo1* (*NBE.6.06A*)	377.36	26.66	5.14	3.79e-104	4.69e-005	∙		∙		∙	AY967650	15866156
*Smed-lin9*	47.51	13.76	2.57	9.25e-012	5.19e-003	∙			∙	∙	KM981930	
*Smed-maf*	19.50	7.45	0	1.91e-006	7.81e-003	∙	∙	∙			KM981931	
*Smed-med7*	162.86	11.18	7.70	3.59e-038	1.44e-001	∙					KM981932	
*Smed-med27*	72.51	14.91	5.14	6.99e-017	1.48e-002	∙					KM981933	
*Smed-meis-like*	10.99	0	0	4.88e-004	1	∙	∙	∙		∙	KM981934	
*Smed-mlx*	160.12	0	40.23	1.81e-017	9.09e-013	∙	∙		∙		KM981935	
*Smed-ncapD2*	84.86	0	0.86	1.11e-024	5.00e-001				∙		KM981936	
*Smed-nfx1 ∥ Smed-stc*	28.29	0.57	0	3.73e-009	5.00e-001	∙	∙	∙			KM981937	
*Smed-nf-YA*	31.31	1.15	2.57	3.48e-007	2.50e-001	∙	∙	∙	∙	∙	KM981938	
*Smed-nf-YB-2*	17.03	0	0	7.63e-006	1	∙	∙	∙	∙	∙	KM981939	
*Smed-nf-YC*	589.38	97.74	142.93	4.54e-064	1.46e-002	∙	∙	∙	∙	∙	KM981940	
*Smed-nme1 ∥ Smed-nm23H1*	603.39	45.00	129.24	4.69e-073	6.54e-010			∙		∙	KM981941	
*Smed-nup50*	45.32	8.89	0.86	6.54e-013	9.77e-003			∙			KM981942	
*Smed-pes1* (*Smed-pescadillo-1*)	228.23	46.15	18.83	5.45e-046	3.34e-004			∙	∙	∙	JX010566	22549959
*Smed-rack1*	115.90	30.10	0	1.91e-033	9.31e-010			∙	∙	∙	KM981943	
*Smed-ranbp2 ∥ Smed-nup358*	45.32	0.86	0.86	6.54e-013	5.00e-001					∙	KM981944	
*Smed-rbbp4-2* (*Smed-rbbp-1*)	100.24	0	0	1.08e-028	1	∙				∙	JX010613	22549959
*Smed-rbbp4-3* (*NBE.6.02C*)	254.04	27.52	17.97	7.94e-054	4.01e-002	∙				∙	AY967644	15866156
*Smed-rbbp4-4*	56.30	6.02	0	1.39e-017	1.56e-002	∙			∙	∙	KM981945	
*Smed-rbbp5*	43.94	0.57	4.28	6.91e-010	1.56e-001	∙		∙		∙	KM981946	
*Smed-rbbp6*	64.27	11.18	0	5.42e-020	4.88e-004					∙	KM981947	
*Smed-rrM2B* (*Smed-rnr2-2*)	826.40	7.45	13.69	1.07e-111	5.54e-002				∙	∙	JX010501	22549959
*Smed-serinc*	12.91	0.29	0	1.22E-004	1						KM981948	
*Smed-set*	936.53	0	63.33	1.70e-100	1.08e-019			∙		∙	KM981949	
*Smed-srrt ∥ Smed-ars2*	249.10	35.54	0	3.75e-073	1.46e-011	∙	∙	∙			KM981950	
*Smed-thoc2*	184.01	9.75	1.71	6.45e-052	1.61e-002			∙		∙	KM981951	
*Smed-tif1A*	63.99	5.73	11.13	1.30e-010	9.44e-002	∙	∙	∙		∙	KM981952	
*Smed-traf-4*	258.16	31.82	0	7.59e-076	2.33e-010	∙				∙	KM981953	
*Smed-traf-5*	47.24	15.19	0	7.11e-015	3.05e-005	∙				∙	KM981954	
*Smed-tsg101* (*NBE.2.10C*)	319.13	164.82	141.22	4.42e-016	3.90e-001	∙		∙	∙	∙	AY967577	15866156
*Smed-tssc1*	48.61	0	8.56	3.69e-008	1.95e-003					∙	KM981955	
*Smed-tusc3*	3.30	0	0	1.25e-001	1			∙		∙	KM981956	

For those genes whose sequence had already been annotated in *S. mediterranea*—although only a slight or no experimental characterization at all had been carried out with them—former gene name is in parentheses and Pubmed identifier of the original publication is provided. When a gene was traditionally known by a different name from the recommended (see Gene nomenclature section in Methods), a synonym name is shown separated by a double bar. Transcription regulators (TR) modulate gene expression. Transcription factors (TF), in addition, posses specific DNA binding domains. Genes involved in embryogenesis/development (ED) and control of cell cycle (CC) are also noted. Oncogenes (OG) have a human homolog related to oncogenesis. Predicted functional domains for several of the selected transcript candidates are visualized in Figure 3.

Although neoblasts are essential also during homeostasis for normal cell renewal, the phenotype becomes more evident during regeneration. Functional analyses were therefore carried out by RNAi followed by head and tail amputation in order to visualize defects in the regenerating process. From the 42 genes whose expression was affected by irradiation, 24 showed a phenotype after RNAi (Additional file 9), most of them preventing a successful regeneration and leading to the death of the animals, the usual phenotype for neoblast genes [58,59].

### New neoblast genes

Interestingly, several of the new genes identified as neoblast genes correspond to transcription factors, which are key elements implicated in cell fate decisions. Furthermore, many are also homologous to cancer related genes. We briefly describe those that produce planarian regeneration impairment after RNAi (Additional file 9). The inhibition of six of them produce a reduced blastema with defective head and eyes. *Smed-atf6A*, is a cyclic AMP-dependent transcription factor, which interacts with the Nuclear Transcription Factor Y (NF-Y) complex (further analyzed later). *Smed-ccar1*, is a perinuclear phospho-protein that functions as a p53 coactivator modulating apoptosis and cell cycle arrest [60]. *Smed-hnrnpA1/A2B1*, a component of the ribonucleosome, is involved in the packaging of pre-mRNA into hnRNP particles in embryonic invertebrate development [61] and in stem cells [62]. *Smed-srrt*, modulates arsenic sensitivity, a carcinogenic compound that inhibits DNA repair [63]. *Smed-med7* and *Smed-med27* belong to a mediator complex essential for the assembly of general transcription factors. *Smed-ranbp2* is a member of the nuclear pore complex and is implicated in nuclear protein import. Within the same family, *Smed-nup50* shows also a stronger phenotype. The knockdown of the other 14 genes prevents the formation of the blastema completely. *Smed-gtf2E1* and *Smed-gtf2F1*, are components of the general transcription factors IIE and IIF. *Smed-ncapD2* is necessary for the chromosome condensation during mitosis [64]. *Smed-pes1*, is required in zebrafish for embryonic stem cell proliferation [65]. *Smed-rack1*, is an intracellular adaptor of the protein kinase C in a variety of signaling processes. *Smed-lin9*, is related to the retinoblastoma pathway interacting with Retinoblastoma 1, which is required for cell cycle progression [66]. All six different retinoblastoma binding proteins produce a non-blastema phenotype. The retinoblastoma pathway has been described to regulate stem cell proliferation in planarians [67] and some of its genes are already identified. Despite that, most of them are yet to be analyzed. Finally, *Smed-rrM2B*, is a subunit of the ribonucleotide reductase (RNR) complex required for DNA repair [68]. Details on these genes as well as the rest of the genes tested from the X1 population can be examined in the Additional file 10.

The four remaining genes presenting an aberrant phenotype during regeneration when inhibited by RNAi are described in detail in the following two sections: the *Smed-meis-like*, a new member of the Meis family, and the three components of the Nuclear Factor Y complex, all of them found to be overexpressed in neoblasts.

### *Smed-meis-like*

*Smed-meis-like* is a member of the TALE-class homeobox family, similar to Meis genes, which was found to be overexpressed in the X1 subpopulation. This gene family is characterized by the presence of a homeobox domain with three extra amino acids between helices 1 and 2 [69]. Some of its members can act as cofactors for *Hox* genes [32]. In *S. mediterranea*, other members of the family have been described: *Smed-prep* [70], *Smed-meis* [54] and *Smed-pbx* [71,72].

**Figure 6:**
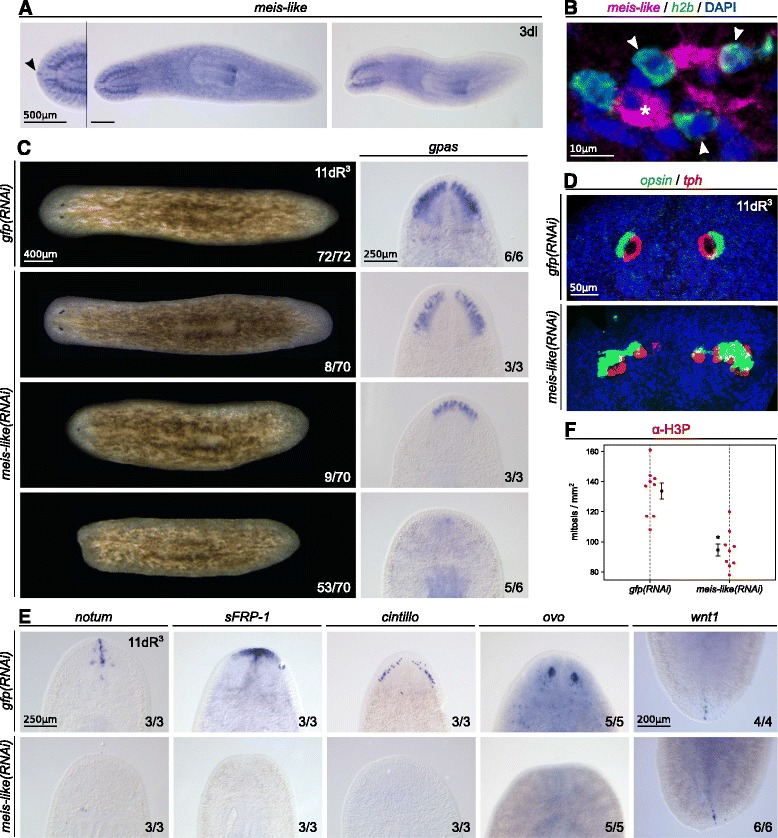
*Smed-meis-like* is essential for anterior regeneration.**A** - WISH reveals that *Smed-meis-like* is expressed in the cephalic ganglia, the pharynx, the tip of the head (arrowhead) and the parenchyma, from where it is downregulated three days after irradiation. **B** - Double FISH of *Smed-meis-like* together with the neoblast marker *Smed-h2b*, shows that *Smed-meis-like* is expressed in neoblasts (arrowheads) as well as in differentiated cells (asterisk). DAPI labels the cell nuclei. See Additional file 11A for the separate channels of fluorescence. **C** - *Smed-meis-like(RNAi)* produce defects in anterior regeneration, which range from an squared head with elongated eyes, cyclops, to complete loss of anterior regeneration. The marker of brain branches *Smed-gpas* also shows this different penetrance. **D** - Double FISH with *Smed-opsin* and *Smed-tph* shows aberrant eyes in the less severe phenotype. **E** - The anterior markers *Smed-notum*, *Smed-sfrp1*, *Smed-cintillo*, and the eye progenitor marker *Smed-ovo* disappear after *Smed-meis-like(RNAi)*, while the posterior marker *Smed-wnt-1* remains. **F** - Quantification of mitotic cells by *α*-H3P immunohistochemistry in the whole animal (*p* < 0.001, *t*-test). All the experiments are done on bipolar regenerating trunks, at 11 days of regeneration after three rounds of injection.

WISH on intact animals shows that it is expressed in the cephalic ganglia, the pharynx, the tip of the head, and the parenchyma (Figure 6A). The downregulation observed three days after irradiation suggests that the parenchyma-associated expression is related to neoblasts and early postmitotic cells. To corroborate this, a double fluorescence in situ hybridization (FISH) together with the neoblast marker *Smed-h2b* [59] has been carried out (Figure 6B and Additional file 11A). Confocal microscopy shows colocalization of both genes in some cells, which confirms the expression of *Smed-meis-like* in neoblasts and, thus, the DGE results. Nevertheless, not all *Smed-meis-like* positive cells are expressing *Smed-h2b*, reinforcing the idea that *Smed-meis-like* is not exclusive of neoblasts.

Knockdown of *Smed-meis-like* through RNAi produced a diverse range of anterior regeneration phenotypes (Figure 6C), which can be explained by a different penetrance. The mildest phenotype produced a squared head with elongated and disorganized eyes. This phenotype was also clearly visible with fluorescence in situ hybridization (FISH) against *Smed-opsin* [5] and *Smed-tph* [73], which label the photoreceptor and the pigment cells of the eye (Figure 6D). In an intermediate phenotype, cyclopic animals are obtained, whereas in the strongest one there is no anterior blastema formation. This range of phenotypes can also be observed with the marker of brain branches *Smed-gpas* [74], which shows a gradual reduction of brain regeneration after *Smed-meis-like* inhibition. These results are also confirmed by the reduction of the brain signal of the pan-neural marker *α*-SYNAPSIN (Additional file 11B). Posterior regeneration was normal.

In the strongest phenotype, there is also no expression of the anterior markers *Smed-notum* [75] and *Smed-sfrp-1* [76,77], and the marker of sensory-related cells *Smed-cintillo* (Figure 6E) [78]. This indicates that *Smed-meis-like* is necessary for anterior identity. In contrast, expression of the posterior marker *Smed-wnt-1* [77] remains after *Smed-meis-like* inhibition. Thus, we can conclude that *Smed-meis-like* is necessary for anterior, but not for posterior regeneration.

Finally, immunohistochemistry against H3P (Figure 6F) shows a slight—but significant—decrease in proliferation in the whole animal (133.8 ±5.22 mitosis/mm^2^ in *n*=9 controls versus 94.6 ±4.06 cells/mm^2^ in *n*=9*Smed-meis-like(RNAi)*, mean ±s.e.m.). This decline in mitosis is matched by the lack of progenitors of some anterior structures, indicating also defects in differentiation. Thus, eye progenitor cells, which are labeled with *Smed-ovo* [54], are not present in *Smed-meis-like(RNAi)* animals (Figure 6E).

The requirement for *Smed-meis-like* in anterior regeneration is similar to another member of the family, *Smed-prep* [70]. This differential phenotype is also observed after the inhibition of other genes, such as *Smed-egr4* [79], *Smed-zicA* [80,81] and *Smed-FoxD* [82]. The milder phenotype, showing elongated eyes, is similar to the effect of *Smed-meis(RNAi)* [54], and also to the mild inhibition of *Smed-bmp4* [83]. Altogether, these results suggest that *Smed-meis-like* is important for eye and anterior regeneration, similarly to other members of the TALE-class homeobox family. However, given the lack of expression of *Smed-meis-like* in the eyes, the abnormal eye formation could be a consequence of the anomalous brain regeneration.

### Nuclear Factor Y complex

The Nuclear Factor Y complex (NF-Y) is an important transcription factor composed by three subunits (NF-YA, NF-YB and NF-YC), each one encoded by a different gene. This heterotrimeric complex acts as both an activator and a repressor, and it regulates other transcription factors, including several growth-related genes, through the recognition of the consensus sequence CCAAT localized in the promoter region [84-88]. In addition, it has been reported that the NF-Y complex regulates the transcription of many important genes like *Hoxb4*, *y-globin*, *TGF-beta receptor II*, or the *Major Histocompatibility Complex class II* and *Sox* gene families [89]. This large number of interactions makes the NF-Y complex an important mediator in a wide range of processes, from cell-cycle regulation and apoptosis-induced proliferation to development and several kinds of cancer [90].

**Figure 7:**
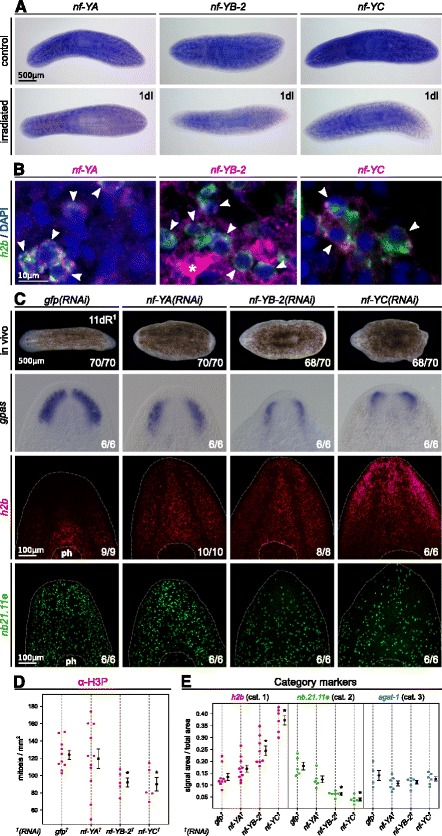
*Smed-nf-Y* gene complex is required for the proper neoblast differentiation and localization.**A** - WISH shows that the three *Smed-nf-Y* genes are expressed ubiquitously and in the cephalic ganglia, and one day after irradiation their expressions decrease. **B** - Double FISH of *Smed-nf-YA*, *Smed-nf-YB-2*, and *Smed-nf-YC* together with the neoblast marker *Smed-h2b* shows colocalization with the NF-Y subunits (arrowheads), demonstrating the expression of this complex in neoblasts as well as in differentiated cells (asterisk). DAPI labels the cell nuclei. See Additional file 12A to check each channel of fluorescence separately. **C** - *Smed-nf-Y(RNAi)* animals regenerate thinner blastemas with non well formed eyes and shape defects, and fail to differentiate a proper brain, with reduced cephalic ganglia as revealed with *Smed-gpas*. FISH with the neoblast marker *Smed-h2b* shows an accumulation of neoblasts in the region in front of the eyes while the early progeny marker *Smed-nb.21.11e* reveals a decrease of early postmitotic cells in *Smed-nf-Y(RNAi)* animals. **D** - Immunohistochemistry with the mitotic marker *α*-H3P shows a reduction in the number of mitosis. **E** - Quantification with category markers indicate a significant increase of *Smed-h2b* ^+^ cells in *Smed-nf-YB-2(RNAi)* and *Smed-nf-YC(RNAi)* animals and a significant decrease of *nb.21.11e* ^+^ cells in all of the RNAi animals, whereas *Smed-agat-1* ^+^ cells do not show significant changes (*p* < 0.001, *t*-test). Counts are referred to the whole body. ph: pharynx. All the experiments are done on bipolar regenerating trunks, at 11 days of regeneration after one round of injection.

In the sexual strain of *S. mediterranea*, an NF-YB is necessary to maintain spermatogonial stem cells [91]. We have isolated a different NF-YB subunit (NF-YB-2), and also a member of the other two subunits (NF-YA and NF-YC). WISH shows that the three genes are expressed ubiquitously and in the cephalic ganglia (Figure 7A). Moreover, the expression decrease one day after irradiation indicating a linkage with stem cells, as described in other organisms [92]. Double FISH of each NF-Y subunit together with *Smed-h2b* confirms the expression of this complex in neoblasts and also in some determined cells (Figure 7B and Additional file 12A).

It has been suggested that each NF-Y component could have a specific role [93]. Therefore, to better understand the function of this complex, we knocked down each subunit separately. Although the penetrance varies depending on the subunit inhibited, the phenotype observed after RNAi treatment is the same. In intact non-regenerating animals, RNAi resulted in head regression, ventral curling and, finally, death by lysis (data not shown), as described for other neoblast-related genes [58,59]. After 11 days, head and tail amputated animals failed to regenerate properly, with a smaller brain and fewer brain ramifications as revealed by *Smed-gpas* (Figure 7C) and by *α*-SYNAPSIN (Additional file 12B). Furthermore, we observe an increase in the number of *Smed-h2b*^+^ cells (Figure 7C,E), also in the area in front of the eyes, where there should not be undifferentiated neoblasts, even though mitosis are reduced (Figure 7D). There is also a decrease in the number of early postmitotic cells (*Smed-nb.21.11e*^+^) (Figure 7C,E), whereas late postmitotic cells (*Smed-agat-1*^+^) do not present significant differences (Figure 7E) [53]. These early progeny markers have recently been associated with epidermal renewal [94]. Hence, the accumulation of neoblasts and the decrease of the subepidermal postmitotic population suggest a defect in the early stages of the differentiation process affecting the epidermal linage. The neural lineage may also be compromised according to the atrophied cephalic ganglia.

## Conclusions

This work presents experimental validation of a collection of putative neoblast genes obtained from a DGE assay on cell fractions. As clearly depicted in the splashplot for the comparison of expression levels between X1, X2 and Xin fractions (Figure 5 and Additional file 13A), there are only a few transcripts specific to X2. The plot produced with the data provided by Labbé [14] from their RNA-Seq analysis on X1, X2 and Xin cell fractions for *S. mediterranea* shows a similar pattern (Additional file 13B). Moreover, comparison among the three sets using Pearson and Spearman correlations indicates that X1 and X2 are the most correlated populations (Additional file 14). Following these results, most of the transcripts expressed in X2 are also expressed in X1. Hence, X2 is a heterogeneous population that cannot be transcriptionally differentiated from X1 without a deeper discrimination method. In this regard, the strategy recently applied by van Wolfswinkel and collaborators using the last sequencing technology to obtain the transcriptome of individual cells [94], represents the most promising approach to deciphering the heterogenity of the neoblast progeny.

Randomization simulations also illustrate the specificity of the 21bp tags to detect real transcripts, corroborating previous estimations [29,46,48,49,95,96]. Furthermore, those results reinforce the assumption that most of the non-mapping tags will correspond to real transcripts [46-49], still lacking from reference data sets for this species. Antisense transcription was also detected, confirming previous reports [25,36,49]. Although further analysis will be required to determine whether this could explain a fraction of the “novel” tags, our primary focus was to characterize the canonical protein-coding transcripts. Due to the heterogeneity of this species genome, we would expect some variability-both at sequence and expression arising from individuals (the pool of animals taken for the samples), and cells (as they do not come from a cell culture). This could explain another fraction of tags not mapping onto the reference transcriptomes. Consequently, we were quite strict in the current manuscript to look for exact tag matches, taking into account that one or more mismatches represents a mappability issue even for finished transcriptomes of the quality of human [97] or *Drosophila melanogaster* [98].

DGE has proven to be reliable for transcript quantification and new gene identification in planaria. In this work, we have described a new member of the TALE-class homeobox family, *Smed-meis-like*. Similar to other members of this family, this gene seems to be involved exclusively on anterior polarity determination during regeneration. Given that the expression of this gene is not restricted to neoblasts, its role can also be important in committed cells. Our results with the NF-Y complex suggest that the knockdown of this complex blocks early differentiation of the epidermal and, probably, neural lineages, both belonging to the ectodermal line, generating a neoblast accumulation and deregulation. This effect has been shown in other organisms such as *Drosophila*, in which NF-Y knockout blocks differentiation of R7 neurons through *senseless* [89,99]. The majority of the new neoblast genes reported and validated in this study were found to participate in cell proliferation, cell cycle regulation, embryogenesis or development in other models, and many of them are involved in processes related to cancer. The pathways participating in tumorigenic processes and stem cell regulation are often the same, as has been proposed previously for planarians [100]. These genes are probably fundamental for stem cell maintenance andthe control of proliferation in organisms with the capacity to regenerate [101], thus reinforcing the potential valueof *S. mediterranea* as an in vivo model for stem cell research [102].

Our DGE analysis pointed out a high resemblance among all the transcriptomes available for *S. mediterranea*. We have also shown the redundancy of the transcriptomes currently available for *S. mediterranea* in agreement with Kao [17], together with their incompleteness under the light of the DGE data. Although our results provide a comprehensive comparison among them, it would be desirable to agree on a unique transcriptome to be used by the whole community. To this end, the PlanMine initiative [103] is attempting to obtain consensus among the researchers on an appropriate reference. Nonetheless, the need for a completely sequenced and well-annotated genome remains. The DGE strategy can help in this endeavour, since short sequences can be rapidly projected over the reference genome or the transcriptome, even from different laboratories, in order to improve their annotation [46]. Similarly, DGE allows the data generated to be reassessed as many times as required, as a more complete genome and transcriptome references for this species become available. Hence, the quantitative data provided here by DGE will prove useful in order to recover and annotate more undescribed genes in the future.

## Methods

### Animal samples

Planarians used in this study were from the asexual clonal line of *S. mediterranea* BCN10. Animals were maintained in artificial water and were starved at least seven days prior to experimentation.

### Cell dissociation, cell sorting and RNA extraction

To trigger neoblast proliferation and differentation, two days head and tail regenerating animals were used for the preparation of the libraries. Three animals per library were used in order to obtain the required amount of RNA. Cell dissociation and FACS were carried out as described by Möritz [31] and Hayashi [30]. Briefly, after cell staining with Calcein AM and Hoechst 33342 (Molecular Probes, Life Technologies), one million cells were separated for each population in a FACSAria sorter (Becton Dickinson) at the Scientific and Technological Centers of the University of Barcelona (CCiTUB) cytometry facilities. A representative plot of the cell populations after the sorting can be seen in Additional file 1A. Cells were directly collected in TRIzol LS (Life Technologies) at 4°C and maintained in ice to preserve RNA integrity. RNA extraction followed to obtain 1 *μ*g of total RNA for each library. Quantification of RNA was assessed with a Nanodrop ND-1000 spectrophotometer (Thermo Scientific) and quality check was performed by capillary electrophoresis in an Agilent 2100 Bioanalyzer (Agilent Technologies) prior to librarypreparation.

### DGE sequencing

Unlike RNA-Seq, this method only sequences a short read of a fixed length, named tag, derived from a single site proximal to the 3’-end of polyadenylated transcripts. This short read is later used to identify the full transcript. The number of times that the very same tag has been sequenced—its number of occurrences—is proportional to the abundance of the transcript which it belongs to. Since it only counts one sequence per transcript, its ability to quantify is not affected by the transcript length. For that reason, DGE is better suited for the detection of short transcripts and low expressed genes when compared with RNA-Seq [20-22].

Sequence tag preparation was done with Illumina’s DGE Tag Profiling Kit according to the manufacturer’s protocol as described [104]. In short, the most relevant steps included the incubation of 1 *μ*g of total RNA with oligo-dT beads to capture the polyadenlyated RNA fraction followed by cDNA synthesis. Then, samples were digested with NlaIII to retain a cDNA fragment from the most 3’ CATG proximal site to the poly(A)-tail. Subsequently, a second digestion with MmeI was performed, which cuts 17 bp downstream of the CATG site, generating, thus, the 21 bp tags.

Cluster generation was performed after applying 4pM of each sample to the individual lanes of the Illumina 1G flowcell. After hybridization of the sequencing primer to the single-stranded products, 18 cycles of base incorporation were carried out on the 1G analyzer according to the manufacturer’s instructions. Image analysis and base calling were performed using the Illumina pipeline, where tag sequences were obtained after purity filtering. Generation of expression matrices, data annotation, filtering and processing were performed by using the Biotag software (SkuldTech, France) [104].

Raw sequencing data in FASTQ format as well as processed tag sequences and their associated expressions have been deposited at NCBI Gene Expression Omnibus (GEO) [105] and are accessible through GEO Series accession number GSE51681 [106].

### Comparison of expression data

Tag raw expression was normalized to counts per million (cpm). The statistical value of DGE data comparisons, as a function of tag counts, was calculated by assuming that each tag has an equal chance to be detected, in fair agreement with a binomial law. An internal algorithm allows the comparison between different libraries and measures the significance threshold for the observed variations and p-value calculation (see Mathematical Appendix of Piquemal et al. 2002 [104]).

Different Perl [107] scripts were designed for the subsequent analyses. All of them are available from the web site planarian.bio.ub.edu/SmedDGE.

### Tag mapping

A database with all the possible CATG + 17bp theoretical tag sequences was constructed for each one of the reference data sets. Tags were compared to these databases to identify all perfect matches and, when more than one tag mapped over the same transcript, only the tag closer to the 3’-end was considered. For the genome reference, 2 mismatches were also considered for unmappable tags with the SeqMap mapper [108,109].

In addition, tags were also mapped against a database of 8,662,308 CDS and 5,189 genomic sequences from bacteria directly downloaded from GenBank [110] repositories to check sample contaminations. Only two tags mapped on bacterial transcripts, confirming the purity of our libraries.

For the 3’-UTR prediction, all 23,020 contigs of the transcriptome from Kao et al. 2013 [17], were mapped over the genome using Exonerate 2.2.0 [111] to characterize the putative 3’-UTR ends (poly-A sites were not predicted though). Apart from aligning the transcripts to the genomic contigs, the strand for the longest ORF contained was also considered to ensure proper transcript orientation. For each transcript, 1,000bp upstream and downstream regions around the genomic coordinate for the putative 3’-UTR ends found were considered to retrieve DGE tags (noted as transcripts 3’-end relative position in Additional file 5).

### Libraries and reference sequence data sets randomization

Libraries and reference data sets were randomized using Perl [107] scripts and the Inline::C library to generate analogous sets of random sequences. This method resembles the original data sets in terms of size and nucleotide abundance in comparison with other approximations which generate virtual sequences based on mathematical distributions [49]. 500 and 100 randomizations for each library and data sets respectively were generated. Mapping was performed using cutoffs of 1, 5, 10, 15 and 20 occurrences (Additional file 3).

### Browsing data sets

Mapped tags are also available from the web site through a set of dynamic tables (Figure 4A). They were implemented using the jQuery jqGrid-4.5.2 [112] library, an Ajax-enabled JavaScript control to represent and manipulate tabular data on the web. Those tables summarize the tags along with their mappings on the different transcriptomes publicly available (which were downloaded from the locations cited at the respective papers [10-17]), their correspondence with the Smed454 transcriptome, and their annotation.

The transcriptome browser shown in Figure 4B was initialized with the Smed454 [10] contigs using the GBrowse2 engine [113]. The browser also includeshigh-scoring segment pairs (HSPs) from whole-transcriptome BLAST searches performed over the UniProt database [114] (NCBI BLAST+ 2.2.29 [51] with default parameters), as well as the Pfam [115,116] domains mapped by HMMER—with E-val =1 and domain E-val =1—[117] on the six-frame translations for the contigs sequences. DGE tag sequences—together with the corresponding counts, normalized scores, their ranks, etc.—were uploaded to the GBrowse2 MySQL database, and they are shown in the browser using a customized version of the Bio::Graphics::Glyph::xyplot module.

Functional annotation was projected from the UniProt GO annotations over the homologous Smed454 contig sequences. Two-tailed hypergeometric test, which accounts for significant overrepresented (positive-tail) or under-represented (negative-tail), was performed by comparing the set of GO assigned to transcriptome contigs over-represented on each of the cell fractions against the set of GO annotations for the whole set of contigs. Significance threshold was set to *p* < 10^-5^ and the results are summarized in Additional file 6 for the different cell fraction sets.

### Gene nomenclature

New genes were named following the nomenclature proposed for *S. mediterranea* [118] based on their BLASTx homology—NCBI BLAST+ 2.2.29 [51] with default parameters against the UniProt database [114]—to its human homologous gene according to the official gene name approved by the HUGO Gene Nomenclature Committee (HGNC) [119] whenever possible, and trying to honor the names of other members of the family if they were already stated for *S. mediterranea*. When no significant homology for the corresponding gene was available, its characteristic domain found at the Pfam site [115,116] was used to identify it.

Gene sequences and primers used for cloning are deposited at the GenBank [110] site—see Table 4 for the accession numbers of the sequences.

### Irradiation

For experimental protocols requiring irradiated animals, irradiation was carried out at 75 Gy (1,66 Gy/minute) in a X-ray cabinet MaxiShot 200 (Yxlon Int.) at the facilities of the Scientific and Technological Centers of the University of Barcelona (CCiTUB).

### In situ hybridization

WISH was conducted for gene expression analysis, as previously described [120,121]. Images from representative organisms of each experiment were captured with a ProgRes C3 camera (Jenoptik) through a Leica MZ16F stereomicroscope. Animals were fixed and hybridized at the indicated time points.

### Fluorescence in situ hybridization

For double FISH animals were treated as described elsewhere [122]. Confocal laser scanning microscopy was performed with a Leica SP2.

### Immunohistochemistry

Immunostaining was carried out as described previously [123]. The following antibodies were used: *α*-SYNORF-1, a monoclonal antibody specific for SYNAPSIN, which was used as a pan-neural marker [124] (1:50; Developmental Studies Hybridoma Bank); and *α*-phospho-histone H3 (H3P), which was used to detect mitotic cells (1:500; Cell Signaling Technology). Alexa 488-conjugated goat *α*-mouse (1:400) and Alexa 568-conjugated goat *α*-rabbit (1:1000; Molecular Probes) were used as secondary antibodies.

### RNAi experiments

Double-stranded RNAs (dsRNA) were produced by in vitro transcription (Roche) and injected into the gut of the planarians as previously described [5]. Three aliquots of 32 nl (400-800ng/ *μ*l) were injected on three consecutive days with a Drummond Scientific Nanoject II injector. Head and tail ablation pre- and post-pharyngeally followed the fourth day. If no phenotype was observed after two weeks, a second round of injection and amputation was carried out in the same manner, unless otherwise stated. Control organisms were injected with *gfp* dsRNA.

### Availability of supporting data

All data sets are fully available without restriction. Yet relevant data sets were already included within this article and its additional files, further supporting material, as well as updates, will be publicly available through the project web site [https://planarian.bio.ub.edu/SmedDGE].

Raw sequencing data in FASTQ format, along with processed tag sequences and their associated expressions, have been deposited at NCBI Gene Expression Omnibus (GEO) [105]; they are accessible through GEO Series accession number GSE51681 [106]. Gene sequences and primers used for cloning are deposited at the GenBank [110] repository, the corresponding accession numbers for the gene sequences are listed on Table 4.

## Additional files

Additional file 1 observed. The sensitivity of the cells in these populations to irradiation responds to their composition of neoblasts in different stages of the cell cycle and distinct levels of determination: X1, proliferating stem cells in S/G2/M, and X2, stem cell progeny and proliferating neoblasts in G0/G1. Neoblasts are the only proliferating cells in this organism.

Additional file 2
**Distribution of mapped and orphan tags by number of occurrences.** **A** - Venn diagrams showing the tags overlap between the three cell populations, by occurrence (top), and by significative p-value, (*p* < 0.05, bottom). The number of mapping tags is detailed in italics. **B** - Frequency distribution of tags grouped by its number of occurrences, i.e., sequencing events, in all libraries. Tags detected in a low copy number are prone to be produced by sequencing errors—likely from more abundant tags. As can be appreciated, most of the tags with less than five occurrences do not map over any of the reference data sets, suggesting that those tags are less reliable [49], which is in agreement with the results of the randomization simulations (see the text and Additional file 3). Due to that, tags detected less than five times were discarded in further analysis.

Additional file 3
**Randomization simulations.** Number of tags mapped over the randomized reference data sets, and vice versa, at different occurrences cutoffs. When compared with the theoretical number of matches expected by chance, this facilitates the assessment of the minimum number of counts for a tag to be considered reliable.

Additional file 4
**X1 and X2 in irradiated animals.** Venn diagram showing the overlap between the results presented here and the DGE study conducted over irradiated planarians of the same clonal line by Galloni [36]. The number of mapping tags out of the total is detailed in italics. It can easily be appreciated that most of the tags present in X1 and X2 are not detected by the irradiation approach.

Additional file 5
**Tags potentially mapping in the 3’-UTR regions.** Y-axis represents the number of tags (tag counts) per nucleotide genomic position. The sequenced DGE tags were then classified in two groups: those mapping within the genomic region delimited by the transcript exons (green area), and those mapping outside (blue area). As position 0 depicts the last nucleotide for all the transcripts, we can only observe green marks upstream; blue marks can distribute across all the downstream region too. Background is defined by all those genomic CATG target sequences that do not match to any of the sequenced DGE tags (red areas). Dashed line depicts the average value for the downstream background tag counts.

Additional file 6
**Bar plots of the GO significant terms for different comparisons among X1, X2 and Xin annotation sets.** Each panel presents a list of the significant functional annotations (*p* < 10^-5^, hypergeometric test), along with the corresponding GO code, that are over- or under-represented (computed as log-odds of the term abundance by sequence set) on each of the three ontology domains (Biological Processes, BP; Molecular Functions, MF; and Cellular Components, CC). Bar plots compare results obtained when considering the following four non-overlapping sets: X1-only (red bars), X2-only (green bars), the intersection between X1 and X2 not in Xin (orange), and Xin-only (blue bars). Bars color-filling is proportional to the p-value for the given GO code, thus darker colors corresponds to smaller p-values (all below the significant threshold anyway). A Venn diagram on top of each page represents the comparison made among the fraction sets.

Additional file 7
**Genes involved in stemness, regeneration or tissue homeostasis overexpressed in neoblasts and their progeny.** DGE expression of clones reported in two experimental high-throughput screenings by Reddien [6] and Wenemoser [56] related to regeneration, stemness or tissue homeostasis identified as being overexpressed in neoblasts (*p* < 0.001).

Additional file 8
**Whole mount in situ hybridization of new neoblast genes.** Expression by WISH of new neoblast genes in control (left panel) and irradiated planarians (right panel). 38 out of the 42 genes tested are presented here. The remaining four are characterized in Figures 6A and 7A. Time after irradiation in days is shown in the top right corner for each gene. As expected for neoblast genes, expression is reduced or disappears after irradiation.

Additional file 9
**RNA interference of new neoblast genes showing defects in regeneration.** The stronger and most representative phenotype obtained after RNAi for those new neoblast genes producing aberrant regeneration after head and tail ablation. Days of regeneration and round of injection in superscript, and number of individuals affected with respect to the total are shown in the top right and bottom right corners of each panel. All pictures are dorsal except *Smed-rbbp4-4*, which illustrates the typical ventral curling of dying animals. The inhibition of most of the genes completely prevented the formation of the blastema. For those cases in which a small blastema was allowed to develop, a detail of the anterior part is shown to appreciate the defective head and eyes. For a regenerating control animal see Figures 6C and 7C.

Additional file 10
**Literature review of the new neoblast genes presented in this study.** A description is provided for each one of the new neoblast genes proposed in this study (summarized in Table 4) based on the literature about their homologs in other species.

Additional file 11
**Double fluorescence in situ hybridization of** ***Smed-h2b*** **with** ***Smed-meis-like*** **.** **A** - Double FISH of *Smed-meis-like* together with the neoblast marker *Smed-h2b* shows colocalization of both genes, demonstrating the expression of *Smed-meis-like* in neoblasts. Expression is also detected in differentiated cells. **B** - The pan-neural marker *α*-SYNAPSIN shows the different penetrance of phenotypes of *Smed-meis-like(RNAi)*.

Additional file 12
**Double fluorescence in situ hybridization of** ***Smed-h2b*** **with** ***Smed-nf-YA*** **,** ***Smed-nf-YB-2*** **, and** ***Smed-nf-YC*** **.** **A** - Double FISH of *Smed-nf-YA*, *Smed-nf-YB-2*, and *Smed-nf-YC* shows colocalization of the NF-Y subunits with the neoblast marker *Smed-h2b*, corroborating the expression of this complex in neoblasts. Expression is also detected in differentiated cells. **B** - The pan-neural marker *α*-SYNAPSIN shows reduced cephalic ganglia of RNAi animals compared with *gfp* controls.

Additional file 13
**Splashplot projection of the X1/X2 versus Xin expression changes of upregulated contigs by cell population.** **A** - Splashplot for overrepresented contigs in the three cell fractions X1, X2 and Xin according to our DGE data over the Smed454 transcriptome [10]. **B** - Same representation using the data published by Labbé [14]. Both plots show a similar composition, revealing a low number of transcripts overexpressed specifically in X2/progeny cells.

Additional file 14
**Pearson and Spearman correlations of the normalized expression levels among X1, X2 and Xin.** Diagonal panels show violin plots with the distribution of the normalized expression levels for each of the three cell populations data sets. Panels on the upper diagonal summarize both Pearson (parametric) and Spearman (non-parametric) correlations, along with the p-values and the linear regression model estimates for the pairwise comparison between data sets. On the bottom diagonal panels, for each pair of cell fractions the scatterplots show differences in expression for each DGE tag. Blue dotted line is defined by the intercept and slope values for the linear regression model presented on the corresponding upper panel, confidence interval is drawn as a grey shadow along that regression line. Those tags having a normalized expression value of zero in one or both of the cell types, when considering each pair-wise comparisons, were removed before computing correlations and for the plots. One can notice that X1 and X2 are the more correlated pair of cell fractions, then X2 and Xin, and finally X1 and Xin. Those results match to what would be expected.

## Abbreviations

3’-UTR: Three prime untranslated region
cpm: Counts per million
DGE: Digital gene expression
EST: Expressed sequence tag
FACS: Fluorescence-activated cell sorting
FISH: Fluorescence in situ hybridization
GO: Gene ontology
ORF: Open reading frame
RNAi: RNA interference
TALE: Three amino acid loop extension
WISH: Whole mount in situ hybridization
