# Supplementary figures and images for: Digital gene expression approach over multiple RNA-Seq data sets to detect neoblast transcriptional changes in Schmidtea mediterranea
Source: BMC Genomics. 2015 May 8;16(1):361. doi: 10.1186/s12864-015-1533-1 (PMC4494696; doi:10.1186/s12864-015-1533-1)

A

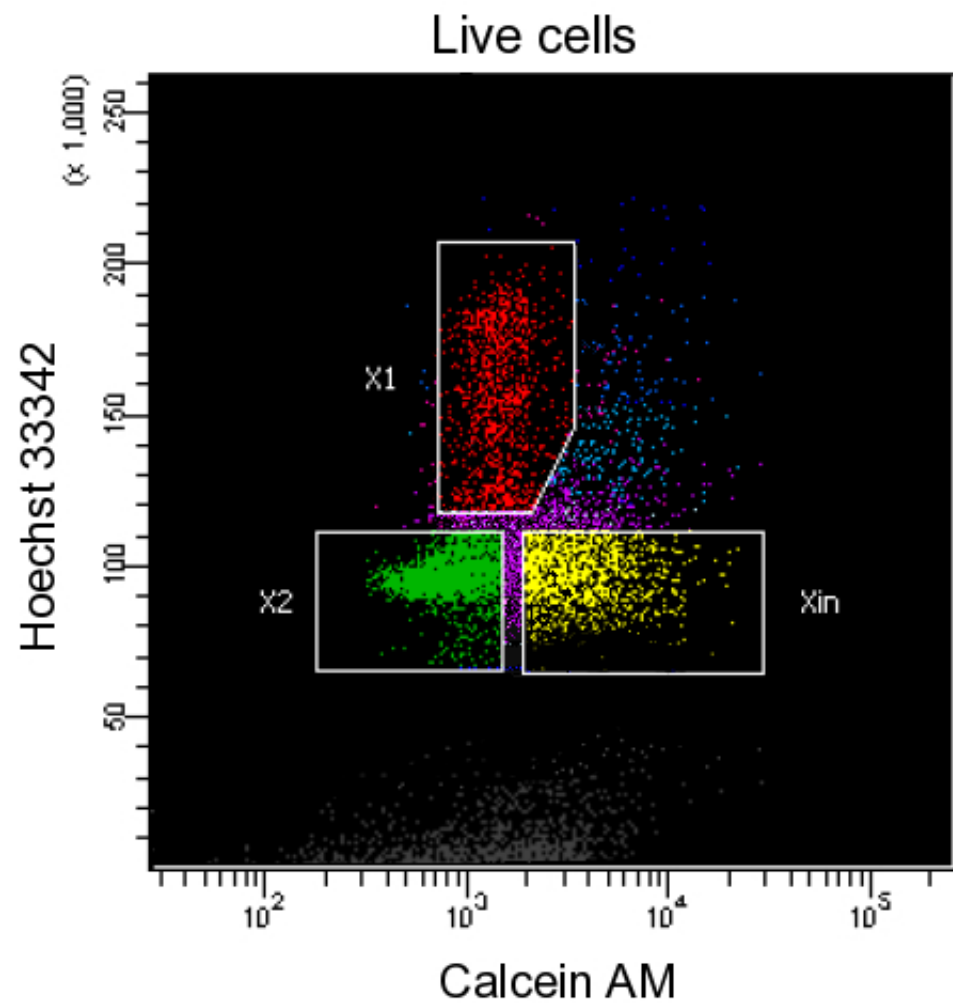

B

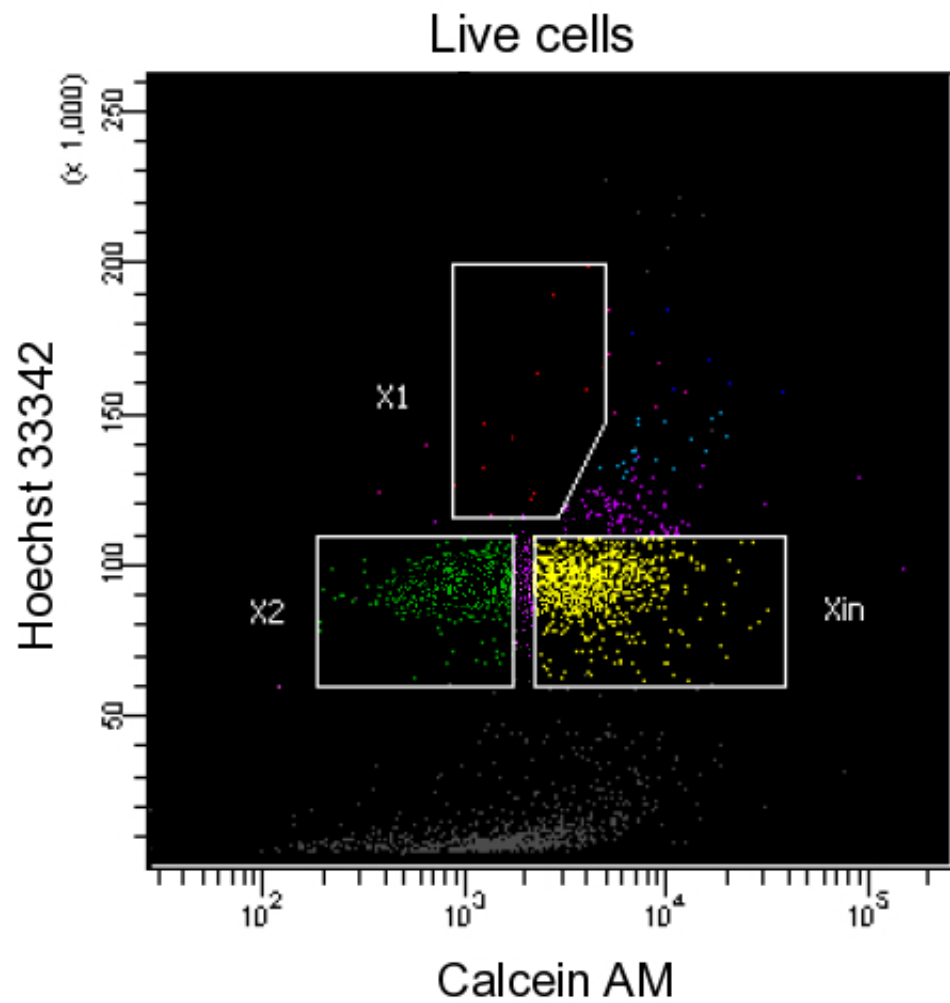

Supplement: Additional file 1 — observed. The sensitivity of the cells in these populations to irradiation responds to their composition of neoblasts in different stages of the cell cycle and distinct levels of determination: X1, proliferating stem cells in S/G2/M, and X2, stem cell progeny and proliferating neoblasts in G0/G1. Neoblasts are the only proliferating cells in this organism. [file 12864_2015_1533_MOESM1_ESM.pdf]

A

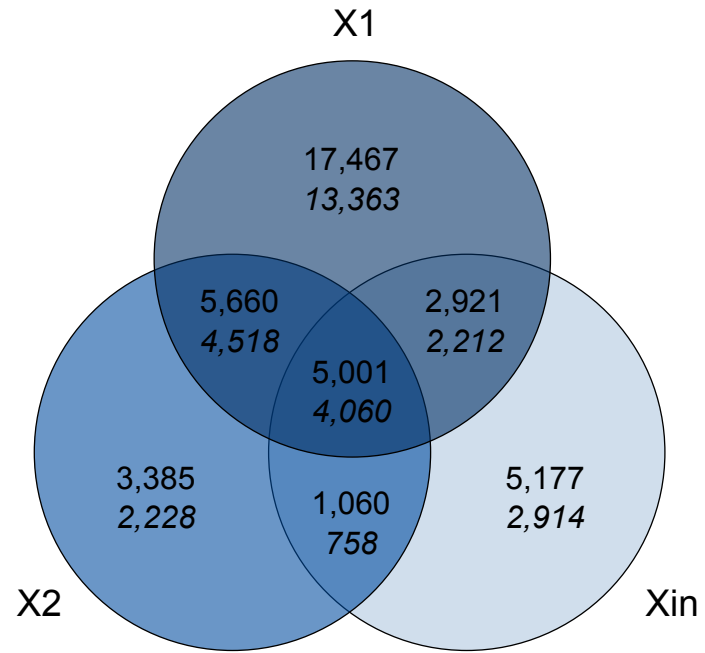

X1 vs Xin

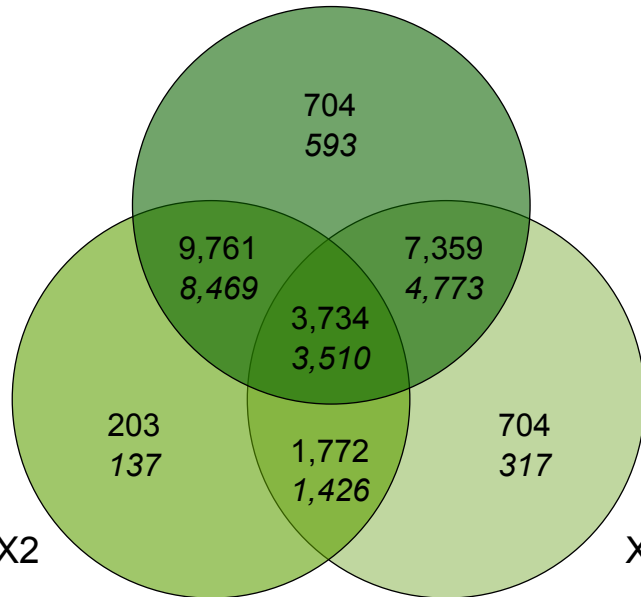

X1 vs X2

X2 vs Xin

B

## TAGs MAPPING

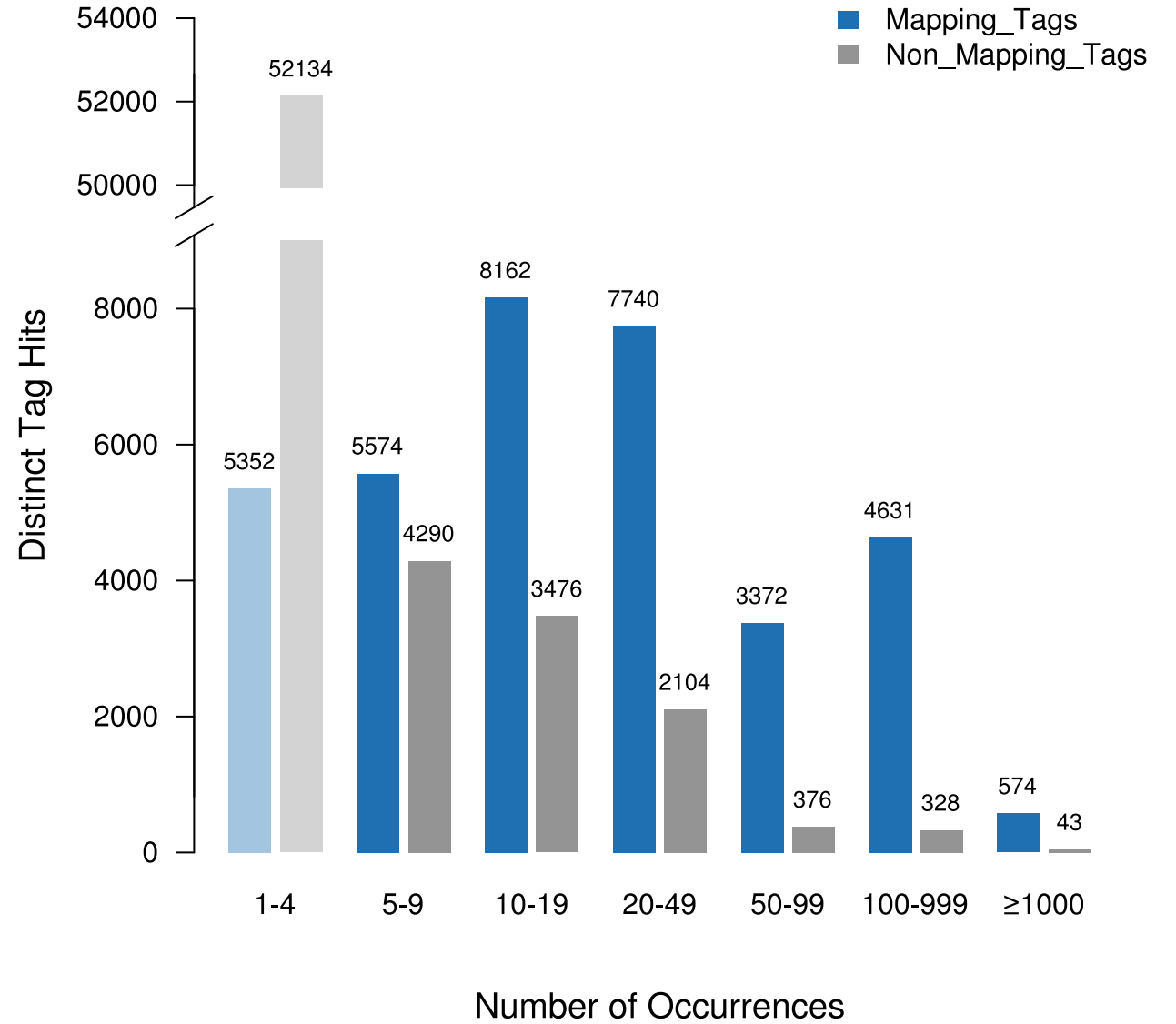

Supplement: Additional file 2 — Distribution of mapped and orphan tags by number of occurrences. A - Venn diagrams showing the tags overlap between the three cell populations, by occurrence (top), and by significative p-value, (p < 0.05, bottom). The number of mapping tags is detailed in italics. B - Frequency distribution of tags grouped by its number of occurrences, i.e., sequencing events, in all libraries. Tags detected in a low copy number are prone to be produced by sequencing errors—likely from more abundant tags. As can be appreciated, most of the tags with less than five occurrences do not map over any of the reference data sets, suggesting that those tags are less reliable [49], which is in agreement with the results of the randomization simulations (see the text and Additional file 3). Due to that, tags detected less than five times were discarded in further analysis. [file 12864_2015_1533_MOESM2_ESM.pdf]

X1 - Xin

15,482

11,838

5,212

4,266

1,985

1,525

448

252

3,129

2,022

256

206

662

357

X2 - Xin

ctrl - irr

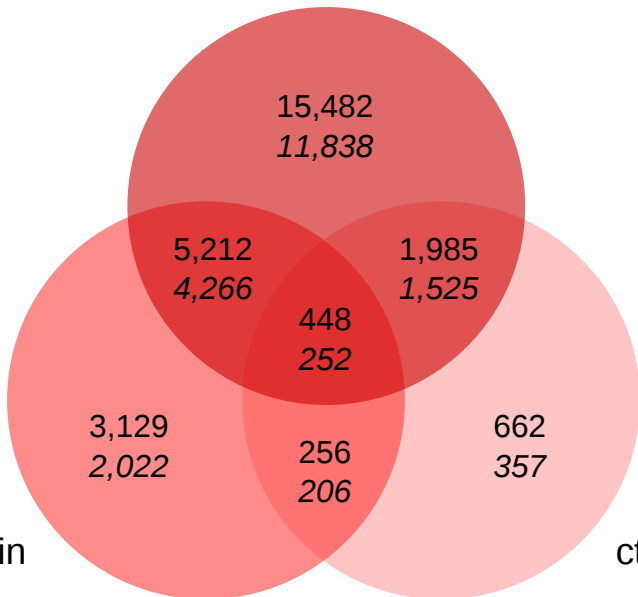

Supplement: Additional file 4 — X1 and X2 in irradiated animals. Venn diagram showing the overlap between the results presented here and the DGE study conducted over irradiated planarians of the same clonal line by Galloni [36]. The number of mapping tags out of the total is detailed in italics. It can easily be appreciated that most of the tags present in X1 and X2 are not detected by the irradiation approach. [file 12864_2015_1533_MOESM4_ESM.pdf]

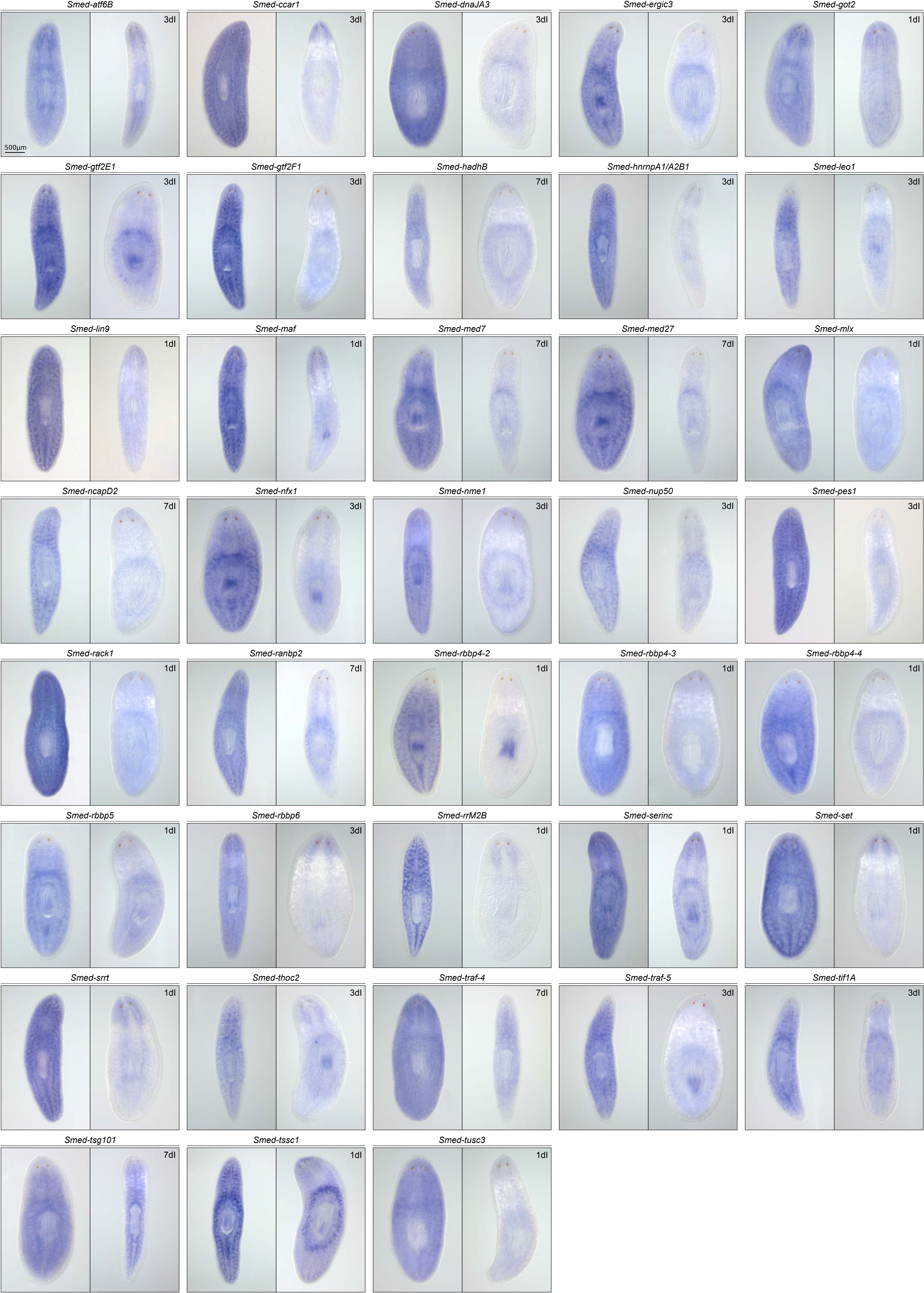

Supplement: Additional file 8 — Whole mount in situ hybridization of new neoblast genes. Expression by WISH of new neoblast genes in control (left panel) and irradiated planarians (right panel). 38 out of the 42 genes tested are presented here. The remaining four are characterized in Figures 6A and 7A. Time after irradiation in days is shown in the top right corner for each gene. As expected for neoblast genes, expression is reduced or disappears after irradiation. [file 12864_2015_1533_MOESM8_ESM.pdf]

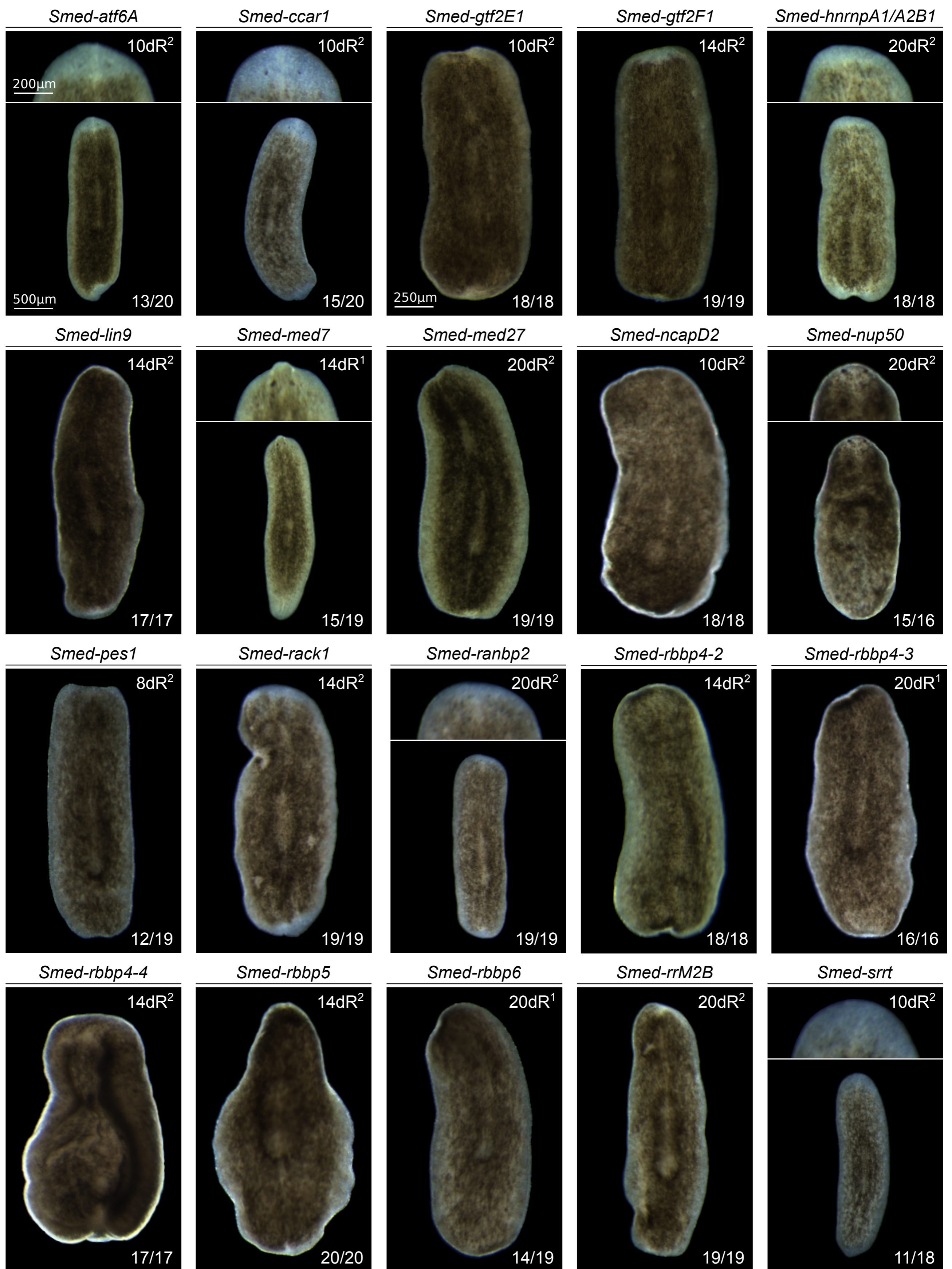

Supplement: Additional file 9 — RNA interference of new neoblast genes showing defects in regeneration. The stronger and most representative phenotype obtained after RNAi for those new neoblast genes producing aberrant regeneration after head and tail ablation. Days of regeneration and round of injection in superscript, and number of individuals affected with respect to the total are shown in the top right and bottom right corners of each panel. All pictures are dorsal except Smed-rbbp4-4, which illustrates the typical ventral curling of dying animals. The inhibition of most of the genes completely prevented the formation of the blastema. For those cases in which a small blastema was allowed to develop, a detail of the anterior part is shown to appreciate the defective head and eyes. For a regenerating control animal see Figures 6C and 7C. [file 12864_2015_1533_MOESM9_ESM.pdf]

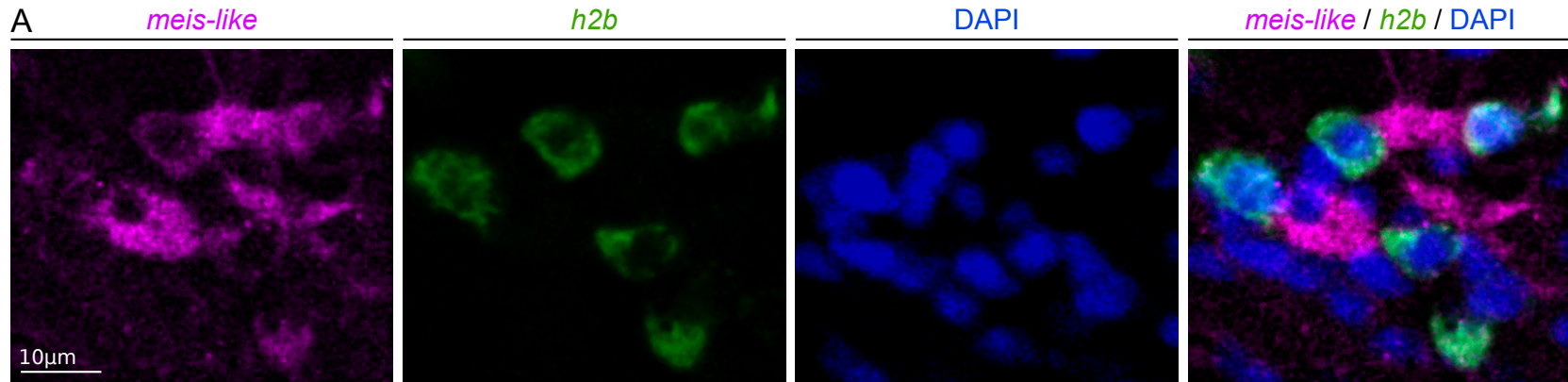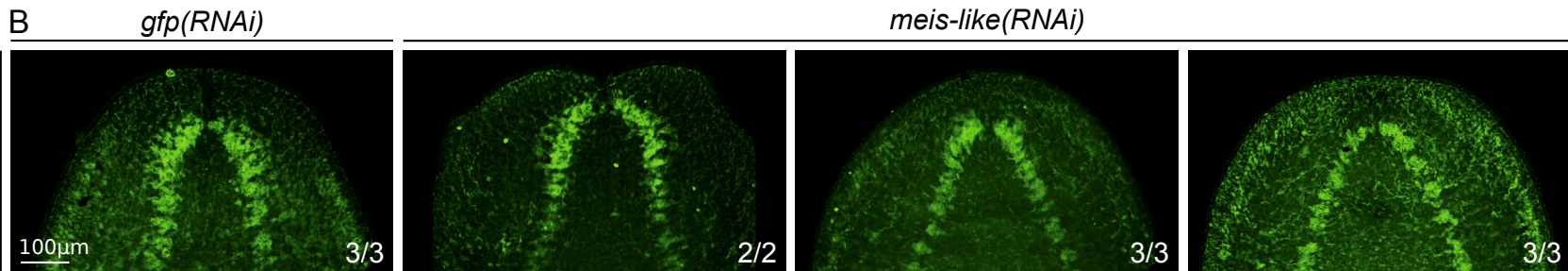

Supplement: Additional file 11 — Double fluorescence in situ hybridization of Smed-h2b with Smed-meis-like . A - Double FISH of Smed-meis-like together with the neoblast marker Smed-h2b shows colocalization of both genes, demonstrating the expression of Smed-meis-like in neoblasts. Expression is also detected in differentiated cells. B - The pan-neural marker α-SYNAPSIN shows the different penetrance of phenotypes of Smed-meis-like(RNAi). [file 12864_2015_1533_MOESM11_ESM.pdf]

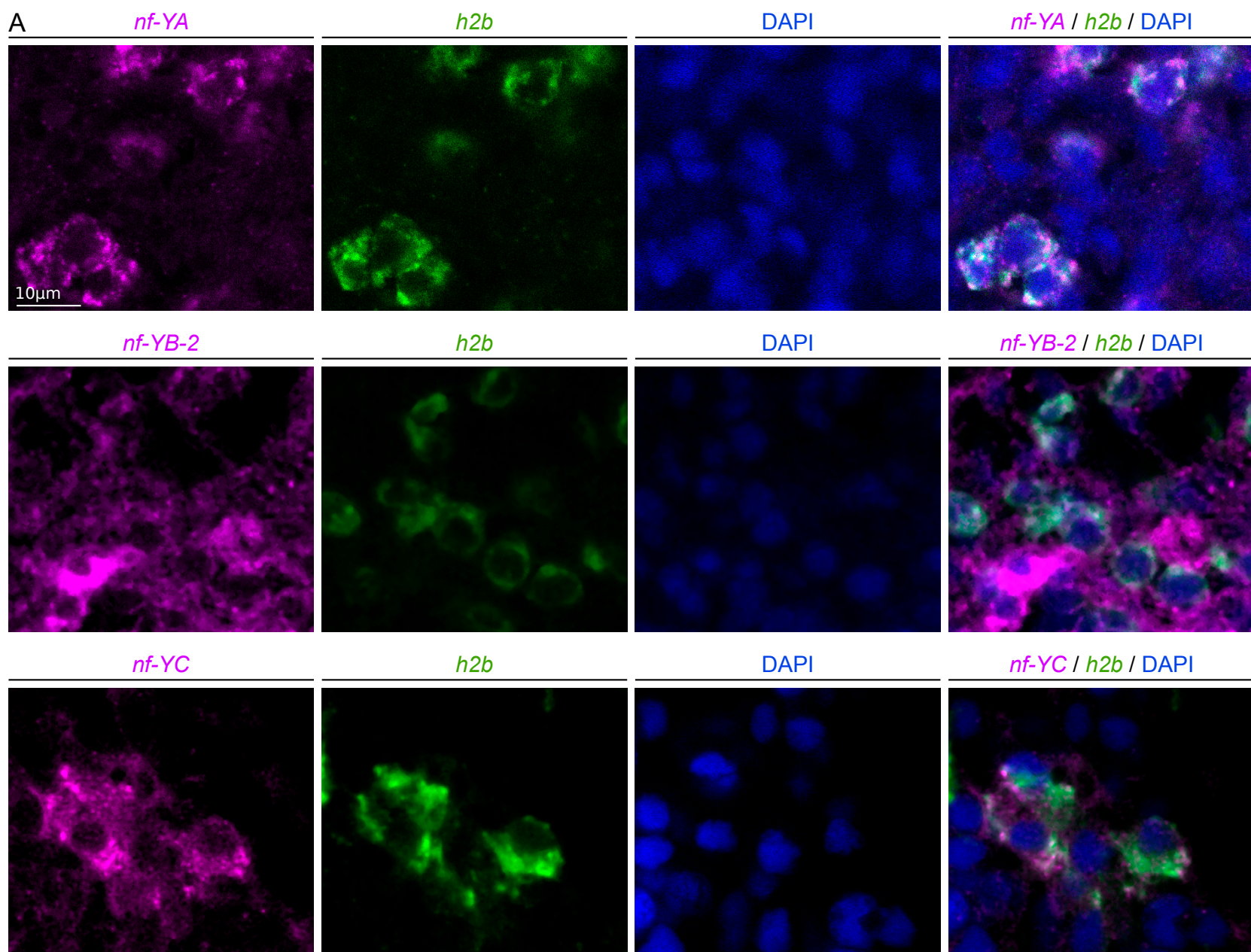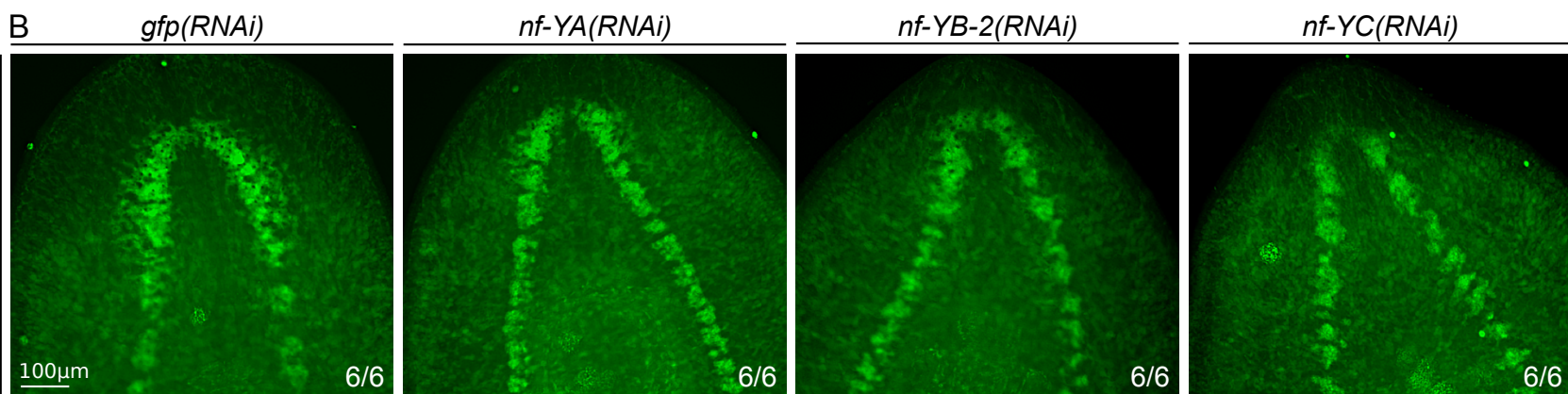

Supplement: Additional file 12 — Double fluorescence in situ hybridization of Smed-h2b with Smed-nf-YA , Smed-nf-YB-2 , and Smed-nf-YC . A - Double FISH of Smed-nf-YA, Smed-nf-YB-2, and Smed-nf-YC shows colocalization of the NF-Y subunits with the neoblast marker Smed-h2b, corroborating the expression of this complex in neoblasts. Expression is also detected in differentiated cells. B - The pan-neural marker α-SYNAPSIN shows reduced cephalic ganglia of RNAi animals compared with gfp controls. [file 12864_2015_1533_MOESM12_ESM.pdf]
