# Supplementary material for: Digital gene expression approach over multiple RNA-Seq data sets to detect neoblast transcriptional changes in Schmidtea mediterranea
Source: BMC Genomics. 2015 May 8;16(1):361. doi: 10.1186/s12864-015-1533-1 (PMC4494696; doi:10.1186/s12864-015-1533-1)
Supplement: Additional file 5 — Tags potentially mapping in the 3’-UTR regions. Y-axis represents the number of tags (tag counts) per nucleotide genomic position. The sequenced DGE tags were then classified in two groups: those mapping within the genomic region delimited by the transcript exons (green area), and those mapping outside (blue area). As position 0 depicts the last nucleotide for all the transcripts, we can only observe green marks upstream; blue marks can distribute across all the downstream region too. Background is defined by all those genomic CATG target sequences that do not match to any of the sequenced DGE tags (red areas). Dashed line depicts the average value for the downstream background tag counts. [file 12864_2015_1533_MOESM5_ESM.pdf]

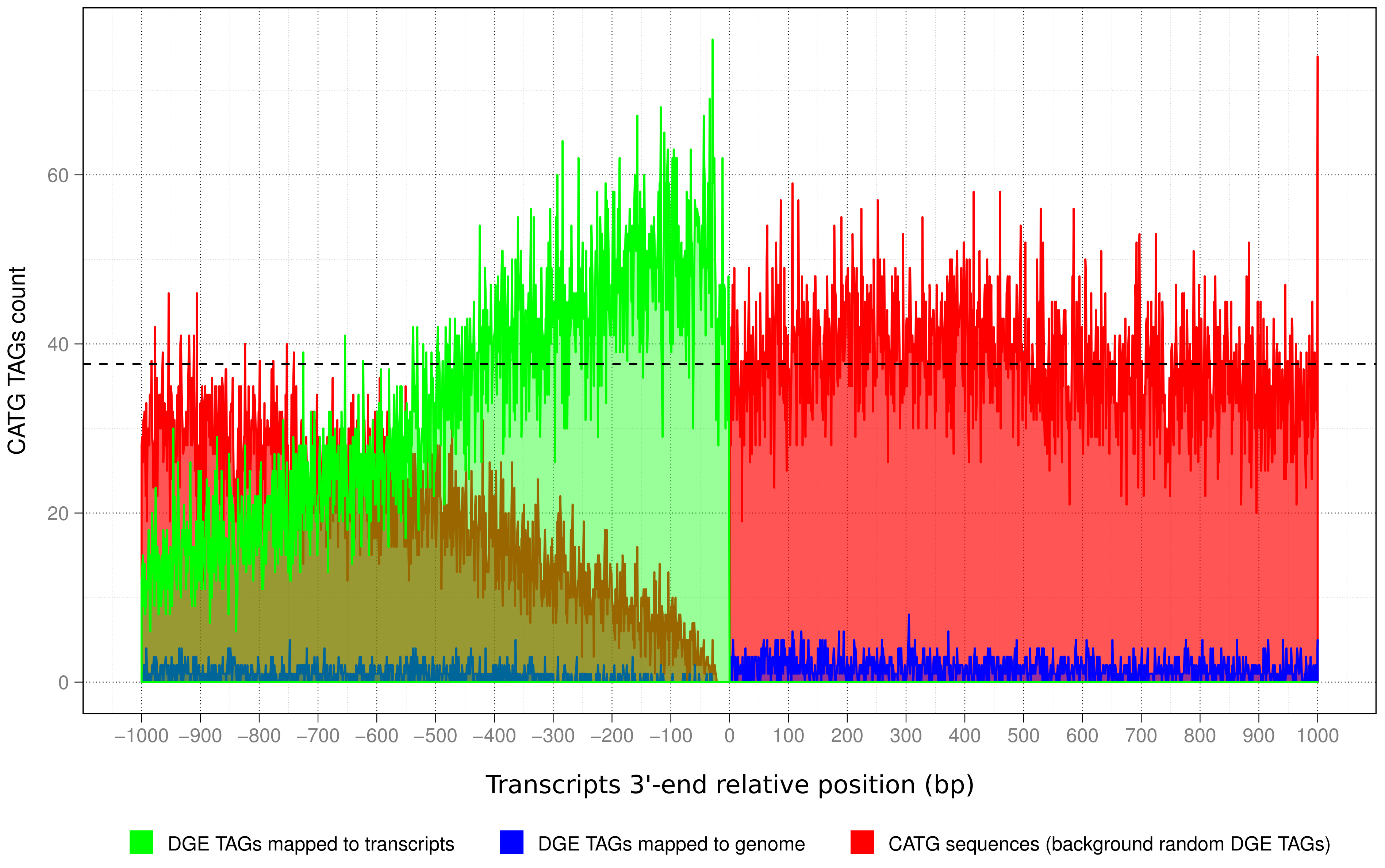

CATG TAGs count
0
20
40
60
−1000
−900
−800
−700
−600
−500
−400
−300
−200
−100
0
100
200
300
400
500
600
700
800
900
1000
Transcripts 3'-end relative position (bp)
DGE TAGs mapped to transcripts
DGE TAGs mapped to genome
CATG sequences (background random DGE TAGs)
