# Supplementary material for: Digital gene expression approach over multiple RNA-Seq data sets to detect neoblast transcriptional changes in Schmidtea mediterranea
Source: BMC Genomics. 2015 May 8;16(1):361. doi: 10.1186/s12864-015-1533-1 (PMC4494696; doi:10.1186/s12864-015-1533-1)

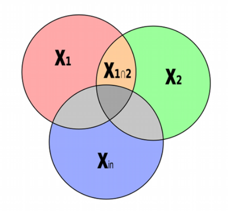

GO BarPlots

$\mathbf{x}_1$ (red) + $\mathbf{x}_2$ (green) + $\mathbf{x}_{1\cap2}$ (orange) + $\mathbf{x}_i$ (blue)

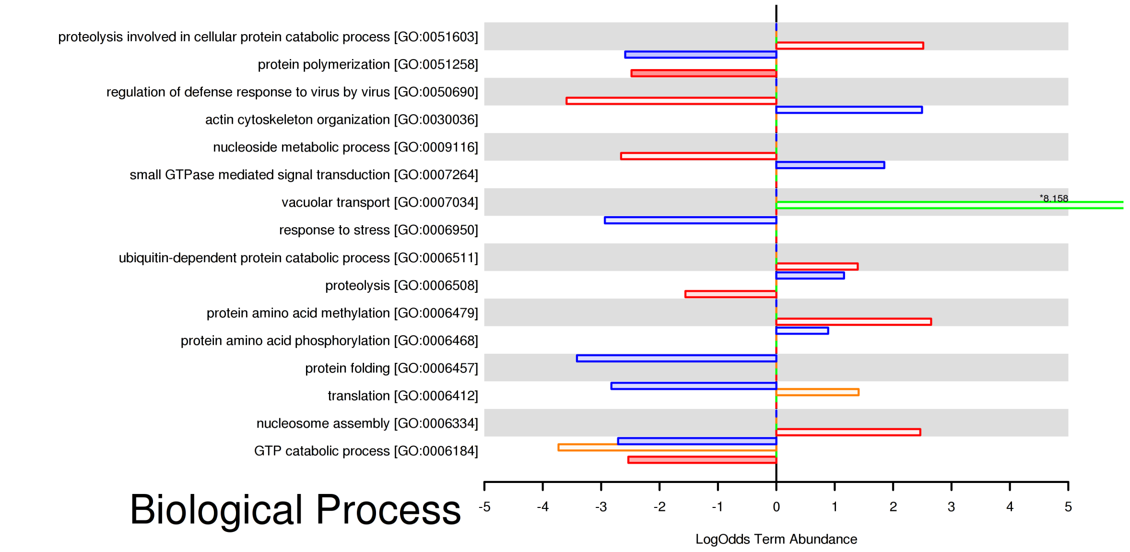

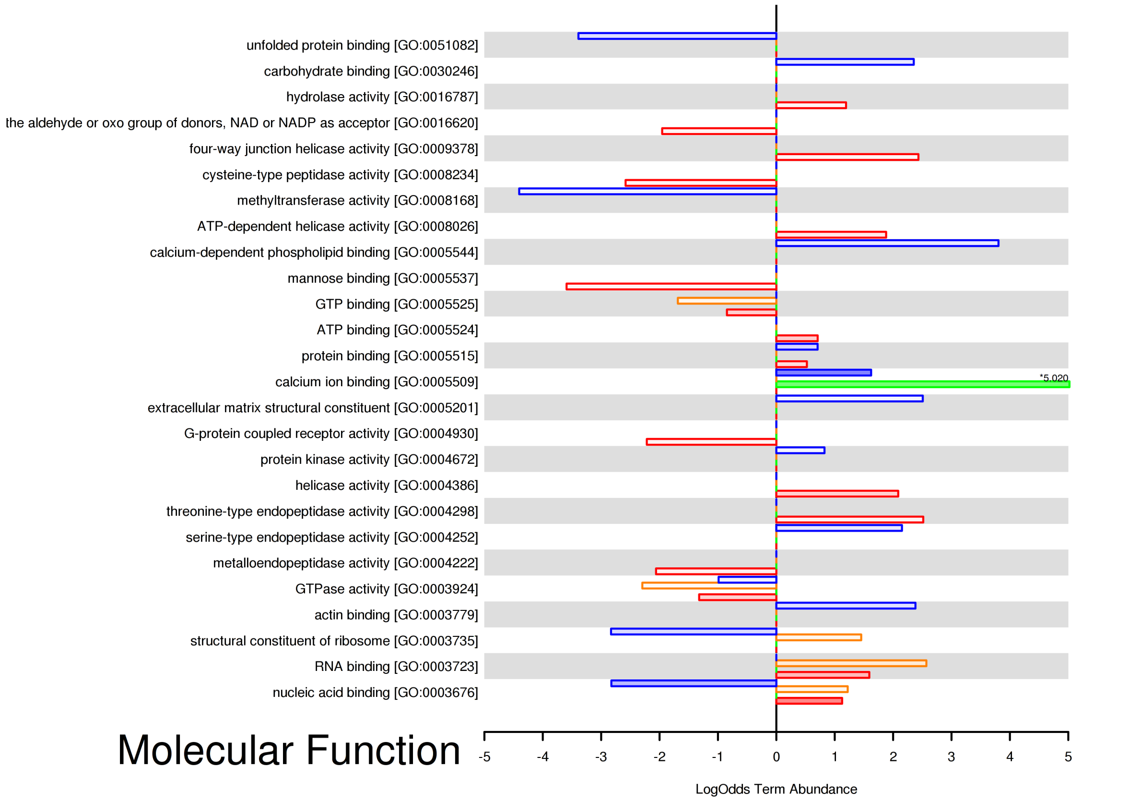

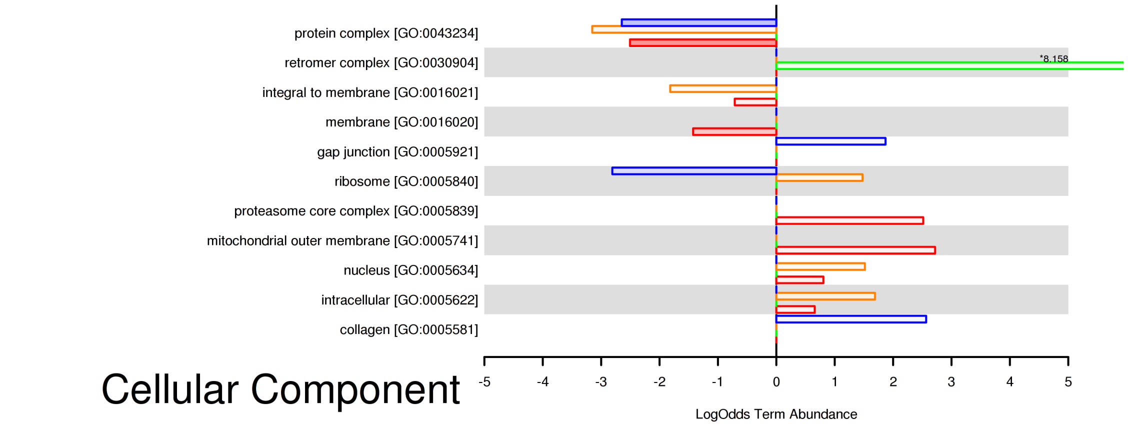

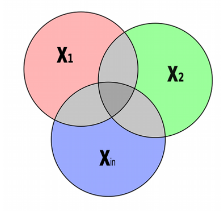

GO BarPlots

x_1 (red) + x_2 (green) + x_i (blue)

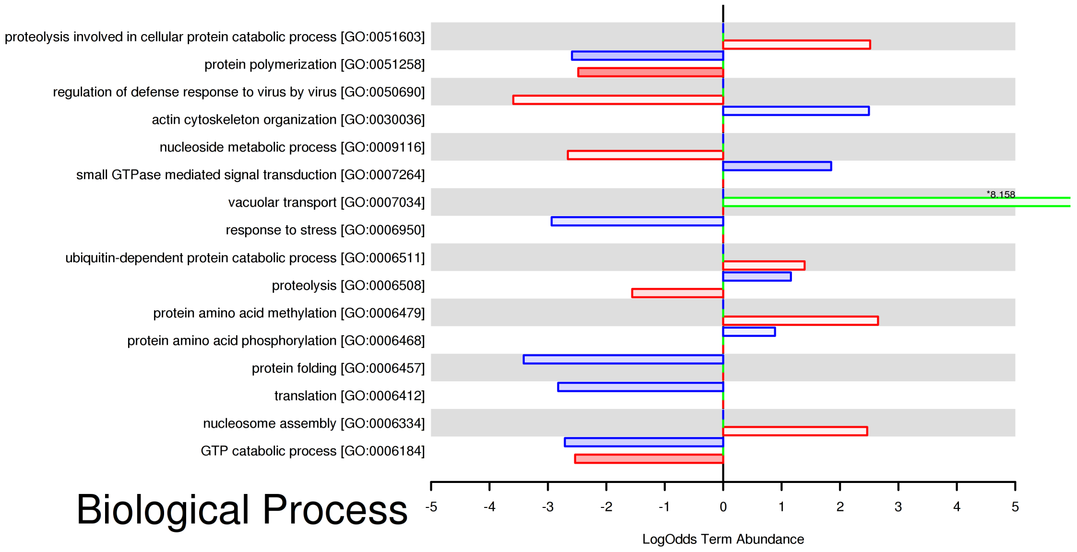

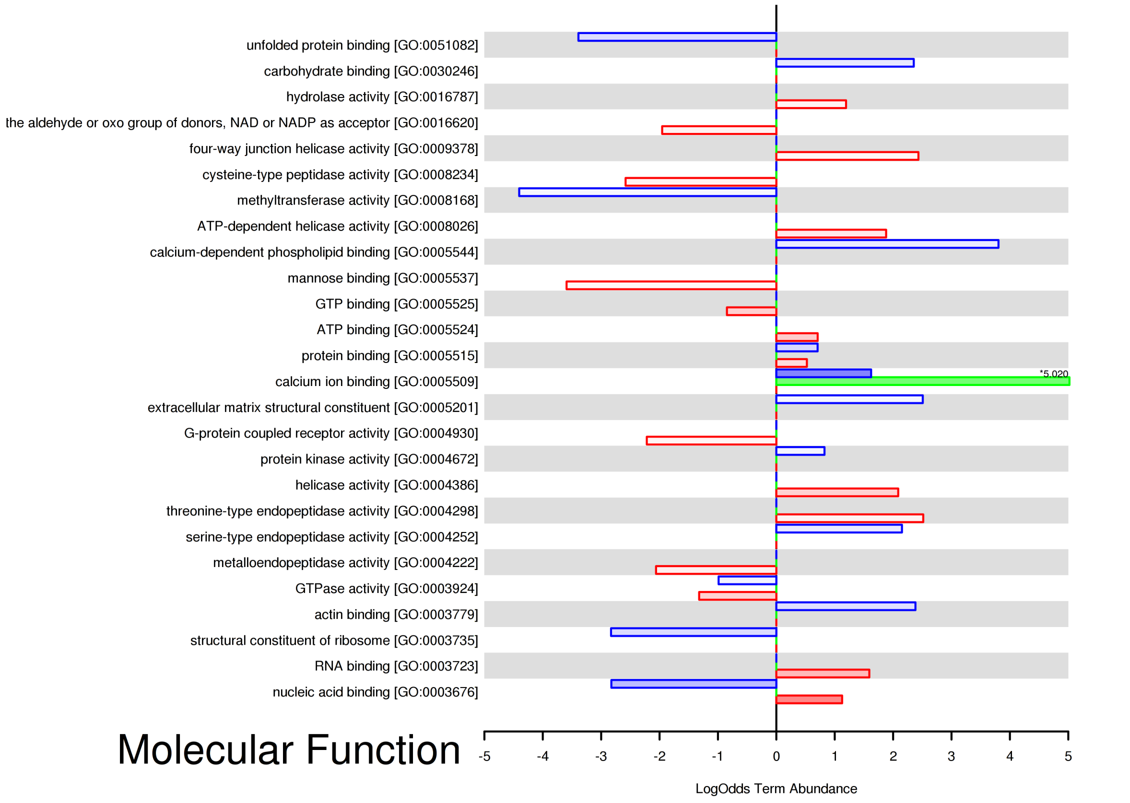

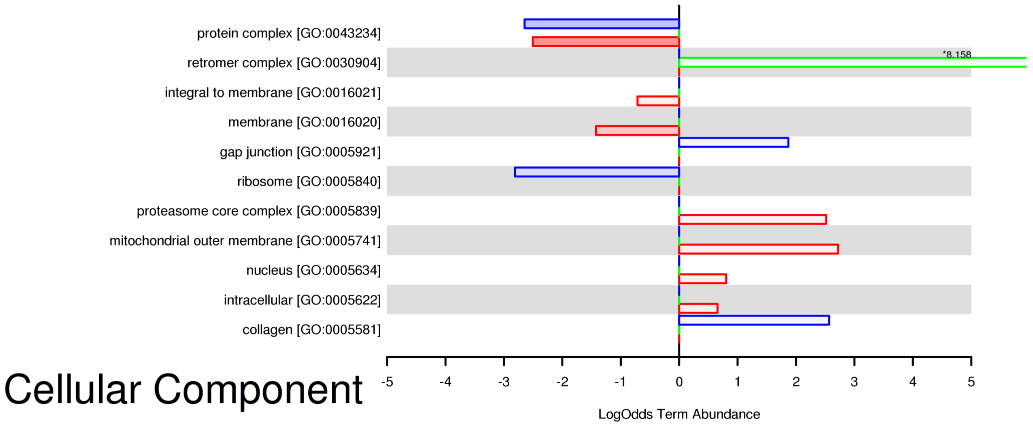

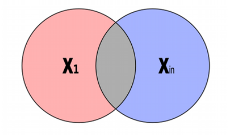

GO BarPlots

x_1 (red) + x_i (blue)

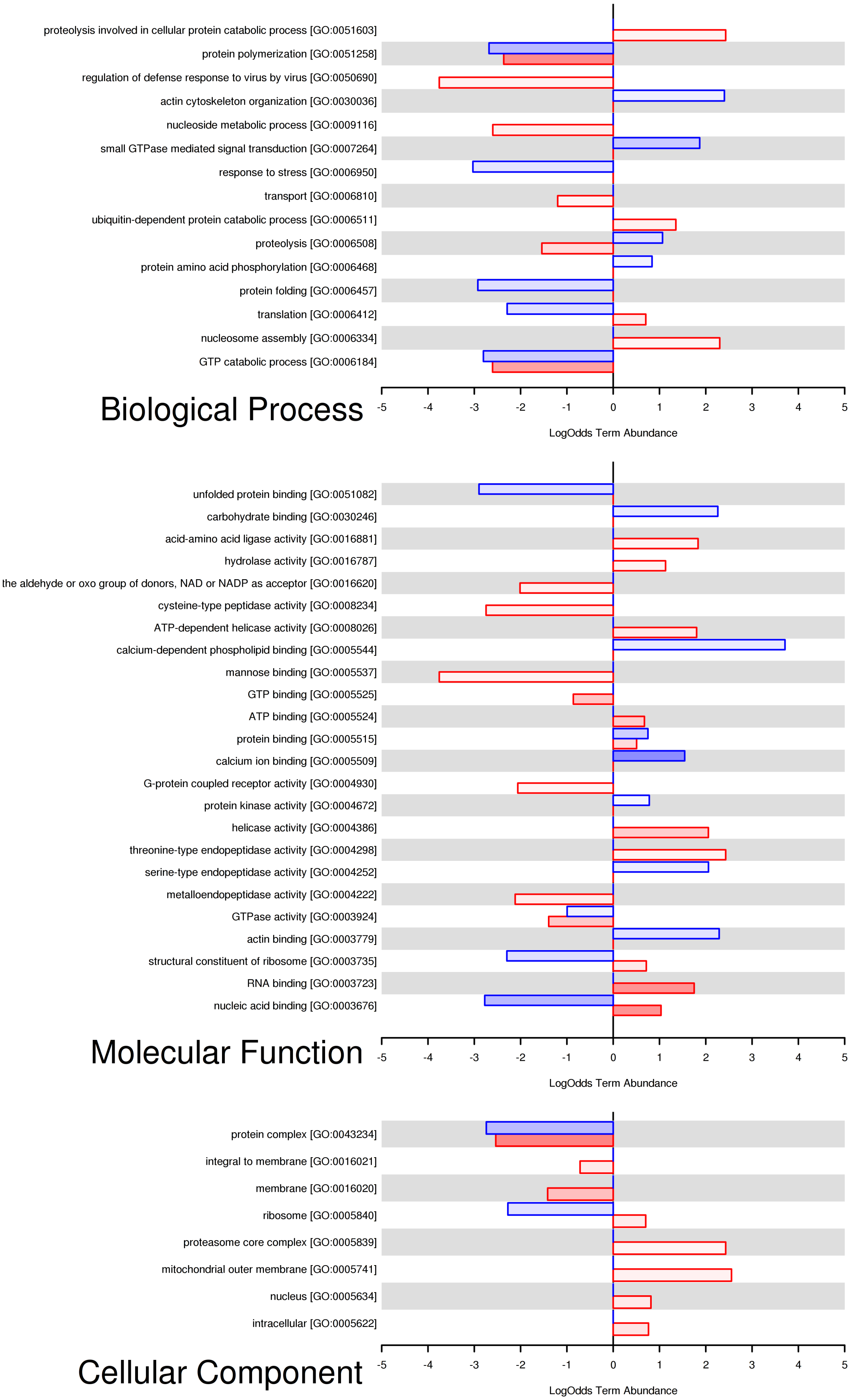

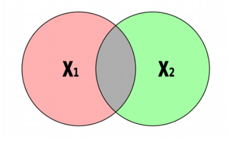

GO BarPlots

x_1 (red) + x_2 (green)

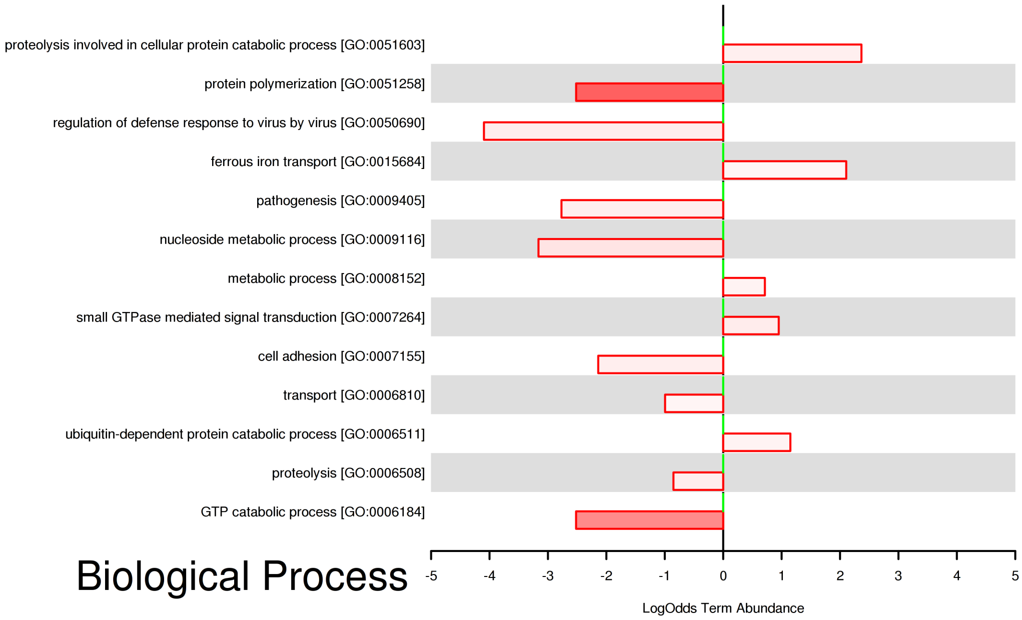

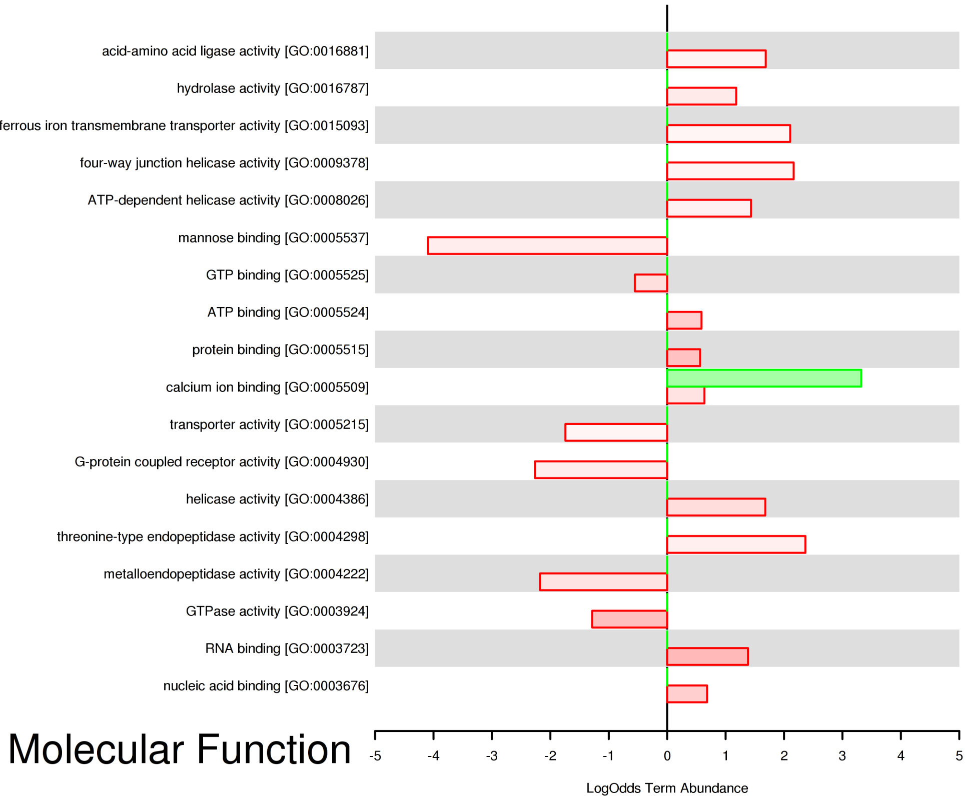

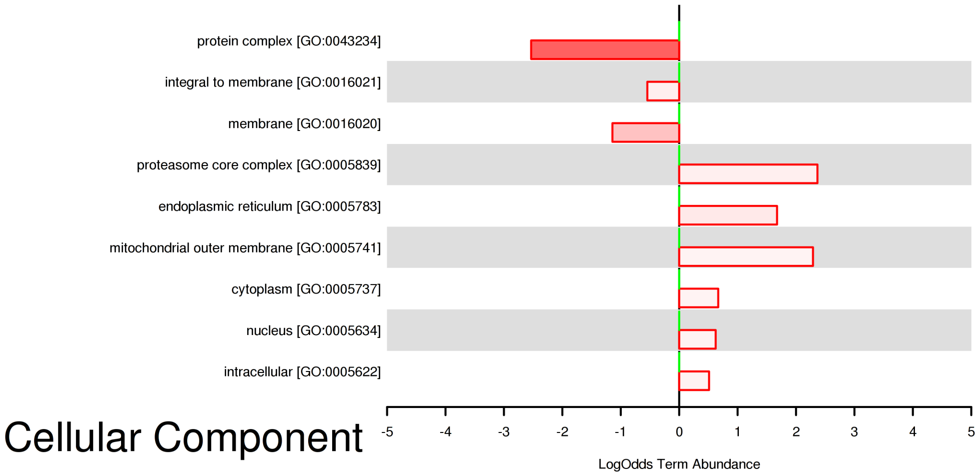

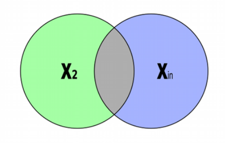

GO BarPlots

x_2 (green) + x_i (blue)

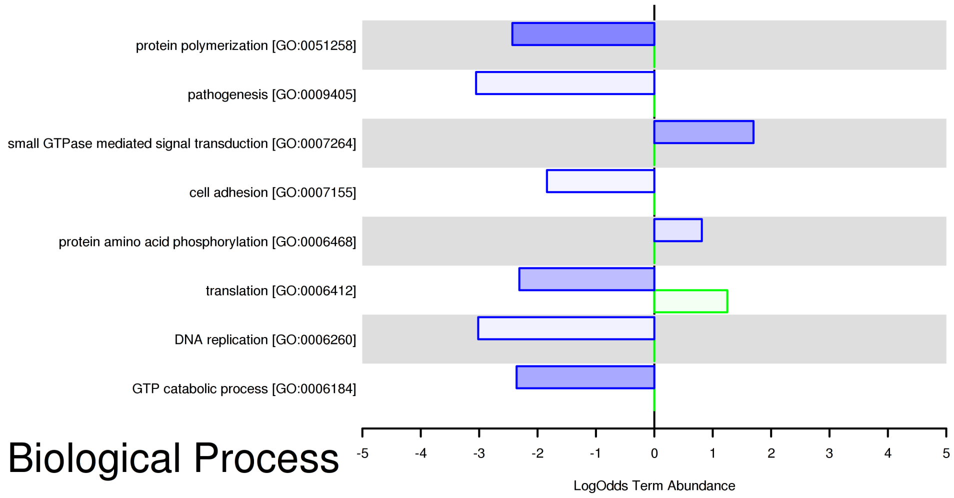

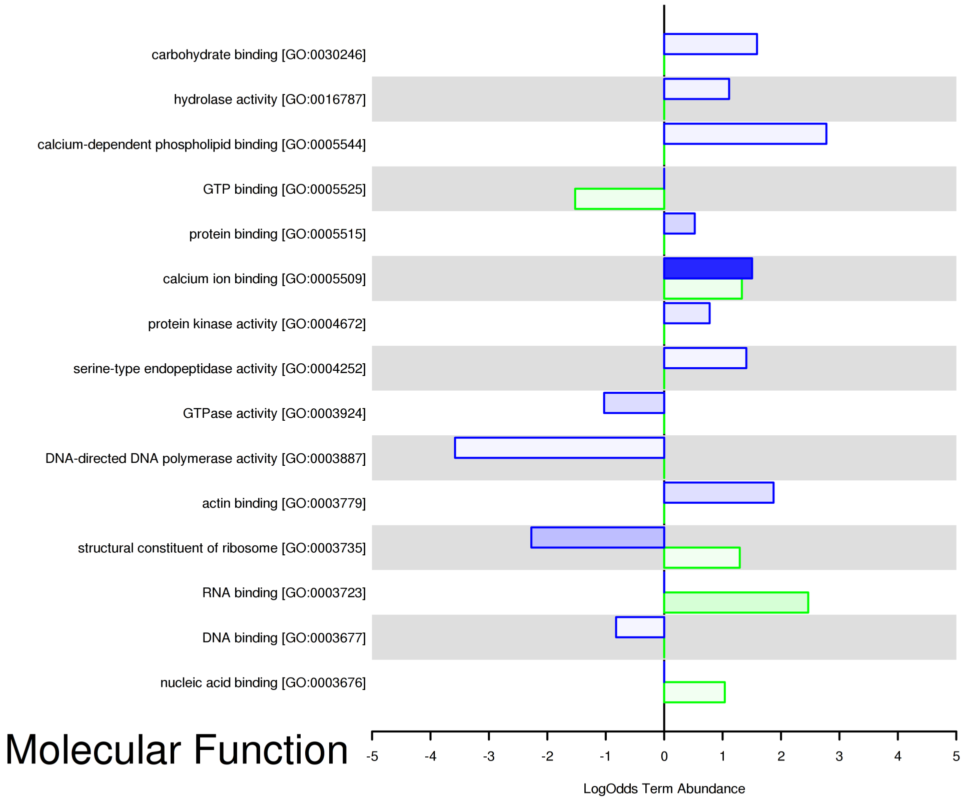

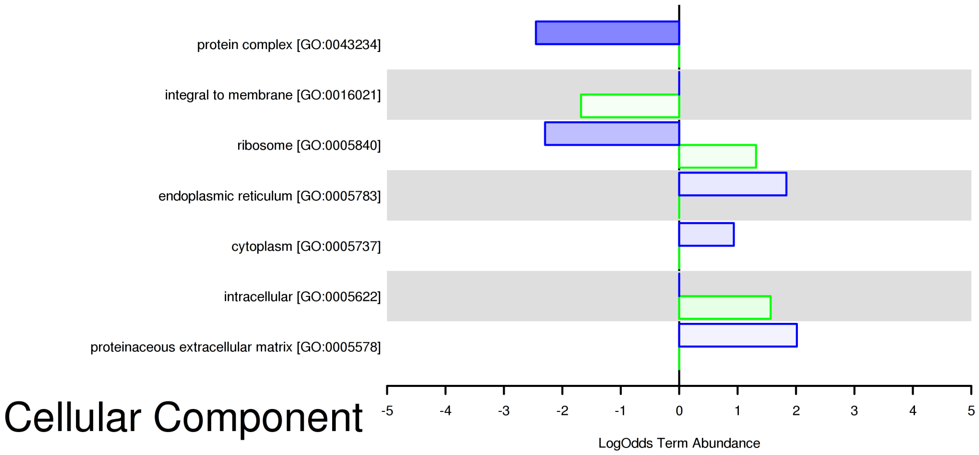

Supplement: Additional file 6 — Bar plots of the GO significant terms for different comparisons among X1, X2 and Xin annotation sets. Each panel presents a list of the significant functional annotations (p < 10-5, hypergeometric test), along with the corresponding GO code, that are over- or under-represented (computed as log-odds of the term abundance by sequence set) on each of the three ontology domains (Biological Processes, BP; Molecular Functions, MF; and Cellular Components, CC). Bar plots compare results obtained when considering the following four non-overlapping sets: X1-only (red bars), X2-only (green bars), the intersection between X1 and X2 not in Xin (orange), and Xin-only (blue bars). Bars color-filling is proportional to the p-value for the given GO code, thus darker colors corresponds to smaller p-values (all below the significant threshold anyway). A Venn diagram on top of each page represents the comparison made among the fraction sets. [file 12864_2015_1533_MOESM6_ESM.pdf]
