# Supplementary material for: Digital gene expression approach over multiple RNA-Seq data sets to detect neoblast transcriptional changes in Schmidtea mediterranea
Source: BMC Genomics. 2015 May 8;16(1):361. doi: 10.1186/s12864-015-1533-1 (PMC4494696; doi:10.1186/s12864-015-1533-1)
Supplement: Additional file 10 — Literature review of the new neoblast genes presented in this study. A description is provided for each one of the new neoblast genes proposed in this study (summarized in Table 4) based on the literature about their homologs in other species. [file 12864_2015_1533_MOESM10_ESM.pdf]

New neoblast genes

- *Smed-atf6A*
  The Cyclic AMP-dependent Transcription Factor ATF-6 alpha forms a dimer which interacts with the Nuclear Transcription Factor Y (NF-Y) trimer through direct binding to the subunit C (NF-YC) (reviewed later in this study). It could also be involved in activation of transcription by the Serum Response Factor [1]. Interestingly, *Smed-srf* [2] is also overexpressed in X1 (Table 2).

- *Smed-ccar1*
  Cell division cycle and apoptosis regulator protein 1 (CCAR1) is a perinuclear phospho-protein that associates with components of the Mediator complex and functions also as a p53 coactivator [3], which has a homolog in *S. mediterranea* [4]. Apart from playing an important role in transcriptional regulation, it modulates apoptosis signaling by CD437, a retinoid that causes cell cycle arrest and apoptosis in a number of cancer cells, and plays a role in cell cycle progression and cell proliferation. Loss of c-Myc sensitizes cells to apoptosis by CCAR1, whereas expression of c-Myc inhibits CCAR1-dependent apoptosis [5]. It has been proposed to act as a tumor growth supressor in a variety of cancers, from breast cancer [6] to B-cell lymphoma [7] or medulloblastoma [8]. In addition, expression of CCAR1 has been detected in the neural crest of vertebrate embryos [9] and osteoblasts [10].

- *Smed-dnaJA3*
  Molecular chaperones are a diverse family of proteins that function to protect proteins during synthesis and from cellular stress. Mitochondrial chaperone DNAJ, also known as Hsp40 (heat shock protein 40kD), is expressed in a wide variety of organisms from bacteria to humans, where it modulates apoptotic signal transduction or effector structures within the mitochondrial matrix and can modulate IFN-gamma-mediated transcriptional activity [11]. Isoform 2 inhibits apoptosis whereas Isoform 1 increases apoptosis triggered by Tumor Necrosis Factor (TNF) and could act as a tumor suppressor [12, 13, 14].

- *Smed-ergic3*
  The Endoplasmic Reticulum-Golgi Intermediate Compartment protein 3 probably mediates transport between endoplasmic reticulum and Golgi. Recently, it has been identified as upregulated in lung cancer [15].

- *Smed-got2*
  The glutamic-oxaloacetic transaminase 2 (GOT2) is an aspartate aminotransferase used by pancreatic cancer cells to convert Gln-derived glutamate (Glu) into α-ketoglutarate (αKG) in the mitochondria to fuel the tricarboxylic acid cycle [16]. In breast cancer, GOT2 could serve as a target for anti-neoplastic agents [17].

- *Smed-gtf2E1* and *Smed-gtf2F1*
  Transcriptional initiation and elongation from RNA polymerase II promoters is positively regulated by the General Transcription Factors IIE and IIF. The Fumble domain in the subunit 1 of GTF2E is required for cell division in *Drosophila*. Mutants lacking Fumble exhibit abnormalities in bipolar spindle organisation, chromosome segregation, and contractile ring formation [18].

- *Smed-hadhB*
  HADHB encodes the beta subunit of the mitochondrial trifunctional protein, which has thiolase activity and catalyzes the last three steps of mitochondrial beta-oxidation of long chain fatty acids. In breast cancer cells, it has been shown to be downregulated by the Estrogen Receptor β (ERβ) [19].

- *Smed-hnrnpA1/A2B1*
  A component of the ribonucleosome, the Heterogeneous Nuclear Ribonucleoprotein A1, A2/B1 homolog, involved in the packaging of pre-mRNA into hnRNP particles has been described to be abundant in early embryo development in insects (*Schistocerca americana*) [20], and human embryonic stem cells [21].

- *Smed-leo1*
  The RNA polymerase-associated protein LEO1 is a component of the PAF1 complex (PAF1C) which has multiple functions during transcription by RNA polymerase II and seems to be required for heart formation and neural crest development in zebrafish (*Danio rerio*) [22]. It is implicated in maintenance of embryonic stem cell pluripotency [23] and in hematopoiesis and promotes leukemogenesis through *Hox* target genes [24].

- *Smed-lin9*
  Protein Lin-9 homolog is related to the retinoblastoma pathway. It acts as a tumor supressor to prevent oncogenic transformation through its association with Retinoblastoma 1 (RB1) in the LIN Complex (LINC) [25], which regulates the expression of genes required for the progression through the distinct phases of the cell cycle [26].

- *Smed-maf*
  Smed-maf contains a Maf domain with an extended homology region (EHR) particular to this family of transcription factors that allows the recognition of longer DNA motifs (Maf recognition elements or MARE) than other leucine zippers. Small Maf proteins may act as competitive repressors of the NF2-E2 transcription factor together with Fos. In mouse, Maf1 plays an early role in axial patterning and defects in these proteins are a cause of autosomal dominant retinitis pigmentosa [27].

- *Smed-med7* and *Smed-med27*
  MED7 and MED27 are components of the Mediator complex, which serves as

a scaffold for the assembly of general transcription factors associated also with RNA polymerase II transcription. They play a role as intermediaries transducing regulatory signals from upstream transcriptional activator proteins to basal transcription machinery at the core promoter. In zebrafish, MED27 is necessary for the development of dopaminergic amacrine cells in the retina and may also negatively regulate the development of rod photoreceptor cells [28].

- *Smed-mlx*
  The Max-like protein X (Mlx) interacts with the Max network of transcriptional factors regulating cell growth, the transition from proliferation to differentiation and apoptosis. Myc and Mad proteins associate with the bHLHZip protein Max to bind specific DNA sequences and regulate the expression of genes important for cell cycle progression. It has been suggested that Mlx may also associate with a subset of the Mad family of transcriptional repressors to antagonize the growth-promoting action of the *Myc* proto-oncogenes, resembling Max in some cell types or cellular stages and controlling the progression through the S phase and differentiation [29, 30]. Hence, Mlx might be important in the sequence of events leading to cell commitment.

- *Smed-ncapD2*
  NcapD2 is a regulatory subunit of the condensin complex that is required for conversion of interphase chromatin into mitotic condensed chromosomes [31]. In interphase cells, the majority of the condensin complex is found in the cytoplasm until mitosis, when most of the complex is associated with chromatin. At the onset of prophase, the regulatory subunits of the complex are phosphorylated by CDK1, leading to condensin's association with chromosome arms and to chromosome condensation [32].

- *Smed-nme1* and *Smed-set*
  The nucleoside diphosphate kinase NME1 was identified because of its reduced mRNA transcript levels in highly metastatic cells, including melanomas, breast and hepatocellular carcinomas, and neuroblastomas [33, 34, 35]. It acts as a suppressor of tumor metastasis and is implicated in cell death. SET is the specific inhibitor of NME1 [36]. Hence, SET acts as a nuclear proto-oncogene and has been described in myeloid leukemia [37] or Wilms' tumor [38]. The exonuclease TREX1 is in the SET complex and acts in concert with NME1 to degrade DNA during cell death [39]. NME1 is required for neural patterning and cell fate determination in the embryonic brain [40], whereas SET is reported to be involved in renal development [38].

- *Smed-nup50* and *Smed-ranbp2*
  Nup50 and RanBP2 (Ran Binding Protein 2), also known as Nup358, are members of the nuclear pore complex, with a direct role in nuclear protein import. Nup50 plays a role in the control of cell proliferation during formation of the neuroepithelium and its deletion causes embryonic lethality

associated with neural tube abnormalities in mouse development [41]. It is also required for cell differentiation [42]. In addition, RanBP2 malfunction has been associated with inflammatory myofibroblastic tumors and other diseases [43, 44, 45, 46].

- *Smed-nfx1*
  The transcriptional repressor NF-X1 binds to the X-box motif of MHC class II genes and silences their expression. It may be an important effector in regulating the duration of the inflammatory response [47]. Moderate expression of the *Drosophila* homolog protein Shuttle Craft (STC) is seen during embryogenesis and subsequent stages of fly development and plays an essential role during late embryonic neurogenesis, where it seems to affect axon guidance [48]. Different human isoforms of NF-X1 have been identified that either induce telomerase activity together with c-Myc through the hTERT promoter (NFX1-123) or repress it (NFX1-91) [49, 50].

- *Smed-pes1*
  Pescadillo was originally identified in zebrafish, where it is widely and dynamically expressed during the first three days of embryogenesis. Its loss blocks the expansion of a number of tissues, generating severe defects in the developing embryo that suggest a role in the control of cell proliferation [51]. It has been confirmed to cause cell cycle arrest in mammals, in which it is essential in the PeBoW-complex for ribosome biogenesis and nucleologenesis during interphase. Blastomeres in mouse embryos lacking pescadillo stop at morula stages of development, the nucleoli fail to differentiate and accumulation of ribosomes is inhibited [52, 53]. Pescadillo is upregulated in malignant cells and may be necessary for oncogenic transformation and tumor progression in certain cancers [54, 55, 56].

- *Smed-rack1*
  RACK1 is a highly conserved intracellular adaptor protein originally identified as the receptor for activated protein kinase C (PKC). It is involved in the recruitment, assembly and regulation of a variety of signaling molecules in many cellular processes: negative regulation of cell growth, positive regulation of cell migration and apoptosis, and regulation of the cell cycle [57, 58]. RACK1 is upregulated in several cancers [59], and plays an important role in angiogenesis and cancer growth [60]. In colon cancer it inhibits cell growth [61] whereas in breast carcinoma it promotes migration and metastasis by interacting with RhoA and activating the RhoA/Rho kinase pathway [62]. It also induces tumorigenicity in lung cancer through activation of the sonic-hedgehog signaling pathway [63]. In *Xenopus* neural development, it is required for neural tube closure [64].

- *Smed-ranbp2*
  See *Smed-nup50* above.

- *Smed-rbbp*
  Up to six different retinoblastoma binding proteins were overexpressed in neoblasts: four *RbbpP4*, one *Rbbp5* and one *Rbbp6* homolog. Transcriptional repression by retinoblastoma is crucial for the proper control of cell growth and it has been reported to regulate stem cell proliferation in freshwater planarians [65]. Three RBBP4s have already been annotated in *S. mediterranea*: Smed-rbbp4-1 has been shown to be expressed in proliferative cells [66] whereas *Smed-rbbp4-2* was not further analyzed or proposed as neoblast gene [2], and *Smed-rbbp4-3* was only described at phenotype level associated with regeneration [67]. RBBP4 is a component of several complexes that regulate chromatin and promote transcriptional repression [68]. Those include the following: Chromatin Assembly Factor 1 (CAF-1), which mediates chromatin assembly in DNA replication and is required for efficient progression through the S phase and may participate in heterochromatin maintenance in proliferating cells [69]; Polycomb Repressive Complex 2 (PRC2), which inhibits homeotic genes during development [70]; and Nucleosome Remodeling and Histone Deacetylase (NuRD), which blocks embryonic stem cell-specific genes [71]. In addition, RBBP4 is associated with cervical cancer (probably through its regulation of tumor supressors p53 and retinoblastoma), thyroid cancer (as a target of Nuclear Factor NF-κB), and apoptosis [72, 73]. The functions of RBBP5 and RBBP6 are less well known. RBBP5 is implicated in acute leukemias as part of the Mixed Lineage Leukemia protein 1 (MLL1) core, which is also essential in embryonic development—where it is predominantly associated with the expression of *Hox* genes [74, 75]. RBBP6 may function as a negative regulator of p53, leading to both apoptosis and cell growth, via MDM2 ubiquitination [76].

- *Smed-rrM2B*
  The M2-B subunit of Ribonucleoside-diphosphate reductase together with the M1 subunit forms an active ribonucleotide reductase (RNR) complex which is expressed in both resting and proliferating cells in response to DNA damage. It also supplies deoxyribonucleotides for DNA repair in a p53-dependent manner in cells arrested at G1 or G2 [77, 78, 79], playing a pivotal role in cell survival, cancer [80, 81, 82] and mitochondrial-associated diseases [83, 84, 85, 86].

- *Smed-serinc*
  Serinc are carrier proteins that incorporate serine into membranes and facilitate the synthesis of lipids derived from this amino acid. They form a unique family of five members with eleven transmembrane domains showing no homology to other proteins but highly conserved in eukaryotes [87].

- *Smed-set*
  See *Smed-nme1* above.

- *Smed-srrt*
  Arsenite is a carcinogenic compound that can act as a comutagen by inhibiting

DNA repair. Serrate RNA effector molecule was firstly described as Arsenite-resistance protein 2 (ARS2), since it modulates arsenic sensitivity [88]. ARS2 controls the multipotent progenitor state of postnatal and adult neural stem cells (NSCs), inducing their self-renewal by direct binding to the promoter of the pluripotency factor *Sox2* and positively regulating its transcription. It may also play a similar role in embryonic stem cells, since ARS2 is also essential for early mammalian development [89, 90].

- *Smed-thoc2*
  As part of the THO Complex, it is required for transcription, processing and nuclear export of spliced mRNA associated with the TREX complex [91]. This complex has been identified as a key element in development, regulating pluripotency and self-renewal of embryonic stem cells, cell differentiation and somatic cell reprogramming as a mature ribonucleoprotein (mRNP) biogenesis factor [92, 93]. It may also be linked to tumorigenesis [94].

- *Smed-tif1A*
  Transcription Intermediary Factor 1 is a transcriptional coactivator with a central role in the regulation of cell proliferation and apoptosis by promoting ubiquitination and proteasomal degradation of p53 [95]. In mice, it functions as a modulator of early embryonic gene expression during the first wave of transcription activation in the zygote [96] and plays a role in the control of retinoic acid-dependent proliferation of hepatocytes, acting as a liver-specific tumor suppressor [97]. In humans, it is known to be upregulated in breast and prostate cancers [98, 99].

- *Smed-traf-4* and *Smed-traf-5*
  TNF Receptor Associated Factors (TRAFs) are a family of scaffold proteins that function as signal transducers of Toll/Interleukin-1 (Toll/IL-1) receptors leading to the activation of the NF-κB transcription factor and the c-Jun N-Terminal Protein Kinases (JNK) among others. Thus, they are involved in the control of inflammation, apoptosis and cell survival. TRAF2 and TRAF3 could act as tumor suppressors, whereas TRAF1 appears to be oncogenic in B cells. Overexpression of TRAF4 and TRAF6 has been reported in breast and lung carcinomas, and in osteosarcoma [100].

- *Smed-tsg101*
  Tumor Susceptibility Gene 101 (TSG-101) takes part in MDM2-p53 regulatory feedback, stabilizing MDM2 and thus promoting p53 degradation through its ubiquitin ligase activity. Hence, loss of TSG101 would result in up-regulation of p53. Stabilization of MDM2 by TSG101 seems to be achieved by inhibiting ubiquitination [101]. This is consistent with the participation of TSG101 in the ubiquitin system as a component of the Endosomal Sorting Complexes Required for Transport 1 (ESCRT-1), which plays a critical function in endosomal sorting and trafficking of ubiquitinated proteins. Despite its name, the involvement of this gene in cancer remains to be clearly demonstrated.

Overexpression of TSG101 has been reported in most cancer types but, while initially discovered as negative regulator for tumorigenesis in a screen for potential tumor suppressors in immortalized fibroblasts, subsequent evidence points out TSG101 as a positive modulator of cancer progression. In any case, TSG101 has also been found to be essential for many processes in the cell, including transcriptional regulation, cell cycle control, and growth and proliferation, and it is clearly required for normal cell function in embryonic and adult tissues [102].

- *Smed-tssc1*
  Tumor Suppressing Subtransferable Candidate 1 (TSSC1) protein was first discovered as one of several tumor-suppressing subtransferable fragments located in the imprinted gene domain of 11p15.5, an important tumor-suppressor gene region [103]. In a recent study it was reported that TSSC1 inhibits breast cancer cell invasion leading to bone metastasis [104];

- *Smed-tusc3*
  Formally a magnesium transporter with a putative function in protein N-glycosylation, Tumor Supressor Candidate 3 gene is closely related with embryonic development and cancer. Morpholino knockdown of TUSC3 protein expression in zebrafish embryos results in early developmental arrest [105]. As a tumor supressor, it has been identified in prostate [106] and ovarian cancer [107] although it has been found expressed in most non-lymphoid cells and tissues examined. In addition, point mutations or deletions in the TUSC3 gene have been identified in individuals with nonsyndromic autosomal recessive intellectual disability (ARID) [108].

References

1. Yoshida, H., Okada, T., Haze, K., Yanagi, H., Yura, T., Negishi, M., Mori, K.: Endoplasmic reticulum stress-induced formation of transcription factor complex ersf including nf-y (cbf) and activating transcription factors 6α and 6β that activates the mammalian unfolded protein response. Mol Cell Biol **21**(4), 1239–1248 (2001)
2. Wenemoser, D., Lapan, S.W., Wilkinson, A.W., Bell, G.W., Reddien, P.W.: A molecular wound response program associated with regeneration initiation in planarians. Genes Dev **26**(9), 988–1002 (2012)
3. Kim, J.H., Yang, C.K., Heo, K., Roeder, R.G., An, W., Stallcup, M.R.: CCAR1, a key regulator of mediator complex recruitment to nuclear receptor transcription complexes. Mol Cell **31**(4), 510–519 (2008)
4. Pearson, B.J., Sánchez Alvarado, A.: A planarian p53 homolog regulates proliferation and self-renewal in adult stem cell lineages. Development **137**(2), 213–221 (2009)
5. Rishi, A.K., Zhang, L., Boyanapalli, M., Wali, A., Mohammad, R.M., Yu, Y., Fontana, J.A., Hatfield, J.S., Dawson, M.I., Majumdar, A.P.N., Reichert, U.: Identification and characterization of a cell cycle and apoptosis regulatory protein-1 as a novel mediator of apoptosis signaling by retinoid cd437. J Biol Chem **278**(35), 33422–33435 (2003)
6. Zhang, L., Levi, E., Majumder, P., Yu, Y., Aboukameel, A., Du, J., Xu, H., Mohammad, R., Hatfield, J.S., Wali, A., Adsay, V., Majumdar, A.P., Rishi, A.K.: Transactivator of transcription-tagged cell cycle and apoptosis regulatory protein-1 peptides suppress the growth of human breast cancer cells in vitro and in vivo. Mol Cancer Ther **6**(5), 1661–1672 (2007)
7. Levi, E., Zhang, L., Aboukameel, A., Rishi, S., Mohammad, R.M., Polin, L., Hatfield, J.S., Rishi, A.K.: Cell cycle and apoptosis regulatory protein (CARP)-1 is a novel, adriamycin-inducible, diffuse large b-cell lymphoma (dlbl) growth suppressor. Cancer Chemother Pharmacol **67**(6), 1401–1413 (2010)
8. Ashour, A.E., Jamal, S., Cheryan, V.T., Muthu, M., Zoheir, K.M.A., Alafeefy, A.M., Abd Allah, A.R., Levi, E., Tarca, A.L., Polin, L.A., Rishi, A.K.: CARP-1 functional mimetics: a novel class of small molecule inhibitors of medulloblastoma cell growth. PLoS ONE **8**(6), 66733 (2013)
9. Adams, M.S., Gammill, L.S., Bronner Fraser, M.: Discovery of transcription factors and other candidate regulators of neural crest development. Dev Dyn **237**(4), 1021–1033 (2008)

10. Sharma, S., Mahalingam, C.D., Das, V., Jamal, S., Levi, E., Rishi, A.K., Datta, N.S.: Cell cycle and apoptosis regulatory protein (CARP)-1 is expressed in osteoblasts and regulated by PTH. Biochem Biophy Res Commun **436**(4), 607–612 (2013)
11. Sarkar, S., Pollack, B., Lin, K., Kotenko, S., Cook, J., Lewis, A., Pestka, S.: hTid-1, a human DnaJ protein, modulates the interferon signaling pathway. J Biol Chem **276**(52), 49034–42 (2001)
12. Schilling, B., De-Medina, T., Syken, J., Vidal, M., Münger, K.: A novel human DnaJ protein, hTid-1, a homolog of the Drosophila tumor suppressor protein Tid56, can interact with the human papillomavirus type 16 E7 oncoprotein. Virology **247**(1), 74–85 (1998)
13. Syken, J., De-Medina, T., Munger, K.: TID1, a human homolog of the Drosophila tumor suppressor l(2)tid, encodes two mitochondrial modulators of apoptosis with opposing functions. Proc Natl Acad Sci **96**(15), 8499–8504 (1999)
14. Tsai, M., Wang, C., Chang, G., Chen, C., Chen, H., Cheng, C., Yang, Y., Wu, C., Shih, F., Liu, C., Lin, H., Jou, Y., Lin, S., Lin, C., Chen, W., Chan, W., Chen, J., Yang, P.: A new tumor suppressor DnaJ-like heat shock protein, HLJ1, and survival of patients with non-small-cell lung carcinoma. J Natl Cancer Inst **98**(12), 825–838 (2006)
15. Wu, M., Tu, T., Huang, Y., Cao, Y.: Suppression subtractive hybridization identified differentially expressed genes in lung adenocarcinoma: ERGIC3 as a novel lung cancer-related gene. BMC Cancer **13**(1), 44 (2013)
16. Son, J., Lyssiotis, C.A., Ying, H., Wang, X., Hua, S., Ligorio, M., Perera, R., Ferrone, C., Mullarky, E., Shyh-Chang, N., Kang, Y., Fleming, J., Bardeesy, N., Asara, J., Haigis, M., DePinho, R., Cantley, L., AC, K.: Glutamine supports pancreatic cancer growth through a KRAS-regulated metabolic pathway. Nature **496**(7443), 101–105 (2013)
17. Thornburg, J., Nelson, K., Clem, B., Lane, A., Arumugam, S., Simmons, A., Eaton, J., Telang, S., Chesney, J.: Targeting aspartate aminotransferase in breast cancer. Breast Cancer Res **10**(5), 84 (2008)
18. Afshar, K., Gönczy, P., DiNardo, S., Wasserman, S.A.: fumble encodes a pantothenate kinase homolog required for proper mitosis and meiosis in *Drosophila melanogaster*. Genetics **157**(3), 1267–1276 (2001)
19. Zhou, Z., Zhou, J., Du, Y.: Estrogen receptor beta interacts and colocalizes with HADHB in mitochondria. Biochem Biophys Res Commun **427**(2), 305–308 (2012)
20. Ball, E.E., Rehm, E.J., Goodman, C.S.: Cloning of a grasshopper cDNA coding for a protein homologous to the A1, A2/B1 proteins of mammalian hnRNP. Nucleic Acids Res **19**(2), 397–397 (1991)
21. Rigbolt, K.T.G., Prokhorova, T.A., Akimov, V., Henningsen, J., Johansen, P.T., Kratchmarova, I., Kassem, M., Mann, M., Olsen, J.V., Blagoev, B.: System-wide temporal characterization of the proteome and phosphoproteome of human embryonic stem cell differentiation. Sci Signal **4**(164), 3–3 (2011)
22. Nguyen, C.T., Langenbacher, A., Hsieh, M., Chen, J.N.: The PAF1 complex component leo1 is essential for cardiac and neural crest development in zebrafish. Dev Biol **341**(1), 167–175 (2010)
23. Ding, L., Paszkowski-Rogacz, M., Nitzsche, A., Slabicki, M.M., Heninger, A.K., de Vries, I., Kittler, R., Junqueira, M., Shevchenko, A., Schulz, H., Hubner, N., Doss, M.X., Sachinidis, A., Hescheler, J., Iacone, R., Anastassiadis, K., Stewart, A.F., Pisabarro, M.T., Caldarelli, A., Poser, I., Theis, M., Buchholz, F.: A genome-scale RNAi screen for Oct4 modulators defines a role of the Paf1 complex for embryonic stem cell identity. Cell Stem Cell **4**(5), 403–415 (2009)
24. Muntean, A.G., Tan, J., Sitwala, K., Huang, Y., Bronstein, J., Connelly, J.A., Basrur, V., Elenitoba-Johnson, K.S.J., Hess, J.L.: The PAF complex synergizes with MLL fusion proteins at HOX loci to promote leukemogenesis. Cancer Cell **17**(6), 609–621 (2010)
25. Gagrica, S., Hauser, S., Kolfschoten, I., Osterloh, L., Agami, R., Gaubatz, S.: Inhibition of oncogenic transformation by mammalian Lin-9, a pRB-associated protein. EMBO J **23**(23), 4627–4638 (2004)
26. Knight, A.S., Notaridou, M., Watson, R.J.: A Lin-9 complex is recruited by B-Myb to activate transcription of G2/M genes in undifferentiated embryonal carcinoma cells. Oncogene **28**(15), 1737–1747 (2009)
27. Kurokawa, H., Motohashi, H., Sueno, S., Kimura, M., Takagawa, H., Kanno, Y., Yamamoto, M., Tanaka, T.: Structural basis of alternative DNA recognition by maf transcription factors. Mol Cell Biol **29**(23), 6232–6244 (2009)
28. Durr, K., Holzschuh, J., Filippi, A., Ettl, A.K., Ryu, S., Shepherd, I.T., Driever, W.: Differential roles of transcriptional mediator complex subunits Crsp34/Med27, Crsp150/Med14 and Trap100/Med24 during Zebrafish retinal development. Genetics **174**(2), 693–705 (2006)
29. Billin, A.N., Eilers, A.L., Queva, C., Ayer, D.E.: Mlx, a novel Max-like BHLHZip protein that interacts with the Max network of transcription factors. J Biol Chem **274**(51), 36344–36350 (1999)
30. Meroni, G., Cairo, S., Merla, G., Messali, S., Brent, R., Ballabio, A., Reymond, A.: Mlx, a new Max-like bHLHZip family member: the center stage of a novel transcription factors regulatory pathway? Oncogene **19**(29), 3266–3277 (2000)
31. Kimura, K., Cuvier, O., Hirano, T.: Chromosome condensation by a human condensin complex in Xenopus egg extracts. J Biol Chem **276**(8), 5417–5420 (2001)
32. Schmiesing, J., Gregson, H., Zhou, S., Yokomori, K.: A human condensin complex containing hCAP-C-hCAP-E and CNAP1, a homolog of xenopus XCAP-D2, colocalizes with phosphorylated histone H3 during the early stage of mitotic chromosome condensation. Mol Cell Biol **20**(18), 6996–7006 (2000)
33. Rosengard, A., Krutzsch, H., Shearn, A., Biggs, J., Barker, E., Margulies, I., King, C., Liotta, L., Steeg, P.: Reduced Nm23/Awd protein in tumour metastasis and aberrant Drosophila development. Nature **342**(6246), 177–180 (1989)
34. Dooley, S., Seib, T., Engel, M., Theisinger, B., Janz, H., Piontek, K., Zang, K., Welter, C.: Isolation and characterization of the human genomic locus coding for the putative metastasis control gene nm23-H1. Hum Genet **93**(1), 63–6 (1994)
35. Carotenuto, M., Pedone, E., Diana, D., de Antonellis, P., Džeroski, S., Marino, N., Navas, L., Di Dato, V., Scoppettuolo, M., Cimmino, F., Correale, S., Pirone, L., Monti, S., Bruder, E., Zenko, B., Slavkov, I.,

Pastorino, F., Ponzoni, M., Schulte, J., Schramm, A., Eggert, A., Westermann, F., Arrigoni, G., Accordi, B., Basso, G., Saviano, M., Fattorusso, R., Zollo, M.: Neuroblastoma tumorigenesis is regulated through the Nm23-H1/h-Prune C-terminal interaction. Sci Rep **3** (2013)
36. Fan, Z., Beresford, P., Oh, D., Zhang, D., Lieberman, J.: Tumor suppressor NM23-H1 is a granzyme a-activated DNase during CTL-mediated apoptosis, and the nucleosome assembly protein SET is its inhibitor. Cell **112**(5), 659–672 (2003)
37. Li, M., Makkinje, A., Damuni, Z.: The myeloid leukemia-associated protein set is a potent inhibitor of protein phosphatase 2a. J Biol Chem **271**(19), 11059–62 (1996)
38. Carlson, S., Eng, E., Kim, E., Perlman, E., Copeland, T., Ballermann, B.: Expression of SET, an inhibitor of protein phosphatase 2A, in renal development and wilms' tumor. J Am Soc Nephrol **9**(10), 1873–80 (1998)
39. Chowdhury, D., Beresford, P., Zhu, P., Zhang, D., Sung, J., Demple, B., Perrino, F., Lieberman, J.: The exonuclease TREX1 is in the SET complex and acts in concert with NM23-H1 to degrade DNA during Granzyme A-mediated cell death. Mol Cell **23**(1), 133–142 (2006)
40. Carotenuto, P., Marino, N., Bello, A., D'Angelo, A., Di Porzio, U., Lombardi, D., Zollo, M.: PRUNE and NM23-M1 expression in embryonic and adult mouse brain. J Bioenerg Biomembr **38**(3-4), 233–246 (2006)
41. Smitherman, M., Lee, K., Swanger, J., Kapur, R., Clurman, B.: Characterization and targeted disruption of murine Nup50, a p27Kip1-interacting component of the nuclear pore complex. Mol Cell Biol **20**(15), 5631–5642 (2000)
42. Buchwalter, A., Liang, Y., Hetzer, M.: Nup50 is required for cell differentiation and exhibits transcription-dependent dynamics. Mol Biol Cell **25**(16), 2472–2484 (2014)
43. Li, J., Yin, W., Takeuchi, K., Guan, H., Huang, Y., Chan, J.: Inflammatory myofibroblastic tumor with RANBP2 and ALK gene rearrangement: a report of two cases and literature review. Diagn Pathol **8**(1), 147 (2013)
44. Chen, S., Lee, J.: An inflammatory myofibroblastic tumor in liver with ALK and RANBP2 gene rearrangement: combination of distinct morphologic, immunohistochemical, and genetic features. Hum Pathol **39**(12), 1854–1858 (2008)
45. Ma, Z., Hill, D., Collins, M., Morris, S., Sumegi, J., Zhou, M., Zuppan, C., Bridge, J.: Fusion of ALK to the Ran-binding protein 2 (RANBP2) gene in inflammatory myofibroblastic tumor. Genes Chromosomes Cancer **37**(1), 98–105 (2003)
46. Pucci, A., Valori, A., Muscio, M., Garofalo, L., Ferroni, F., Abbruzzese, P.: Asymptomatic inflammatory myofibroblastic tumor of the heart: immunohistochemical profile, differential diagnosis, and review of the literature. Cardiovasc Pathol **18**(3), 187–190 (2009)
47. Arlotta, P., Miyazaki, D., Copeland, N.G., Gilbert, D.J., Jenkins, N.A., Ono, S.J.: Murine NFX.1: isolation and characterization of its messenger RNA, mapping of its chromosomal location and assessment of its developmental expression. Immunology **106**(2), 173–181 (2002)
48. Stroumbakis, N.D., Li, Z., Tolias, P.P.: A homolog of human transcription factor NF-X1 encoded by the drosophila shuttle craft gene is required in the embryonic central nervous system. Mol Cell Biol **16**(1), 192–201 (1996)
49. Gewin, L., Myers, H., Kiyono, T., Galloway, D.A.: Identification of a novel telomerase repressor that interacts with the human papillomavirus type-16 E6/E6-AP complex. Genes Dev **18**(18), 2269–2282 (2004)
50. Katzenellenbogen, R.A., Egelkrout, E.M., Vliet-Gregg, P., Gewin, L.C., Gafken, P.R., Galloway, D.A.: NFX1-123 and Poly(A) binding proteins synergistically augment activation of telomerase in human papillomavirus type 16 E6-expressing cells. J Virol **81**(8), 3786–3796 (2007)
51. Allende, M.L., Amsterdam, A., Becker, T., Kawakami, K., Gaiano, N., Hopkins, N.: Insertional mutagenesis in zebrafish identifies two novel genes, pescadillo and dead eye, essential for embryonic development. Genes Dev **10**(24), 3141–3155 (1996)
52. Lerch Gaggl, A., Haque, J., Li, J., Ning, G., Traktman, P., Duncan, S.A.: Pescadillo is essential for nucleolar assembly, ribosome biogenesis, and mammalian cell proliferation. J Biol Chem **277**(47), 45347–45355 (2002)
53. Grimm, T., Hölzel, M., Rohrmoser, M., Harasim, T., Malamoussi, A., Gruber Eber, A., Kremmer, E., Eick, D.: Dominant-negative Pes1 mutants inhibit ribosomal RNA processing and cell proliferation via incorporation into the PeBoW-complex. Nucleic Acids Res **34**(10), 3030–3043 (2006)
54. Kinoshita, Y., Jarell, A.D., Flaman, J.M., Foltz, G., Schuster, J., Sopher, B.L., Irvin, D.K., Kanning, K., Kornblum, H.I., Nelson, P.S., Hieter, P., Morrison, R.S.: Pescadillo, a novel cell cycle regulatory protein abnormally expressed in malignant cells. J Biol Chem **276**(9), 6656–6665 (2000)
55. Prisco, M., Maiorana, A., Guerzoni, C., Calin, G., Calabretta, B., Voit, R., Grummt, I., Baserga, R.: Role of Pescadillo and Upstream Binding Factor in the proliferation and differentiation of murine myeloid cells. Mol Cell Biol **24**(12), 5421–5433 (2004)
56. Maiorana, A., Tu, X., Cheng, G., Baserga, R.: Role of *pescadillo* in the transformation and immortalization of mammalian cells. Oncogene **23**(42), 7116–7124 (2004)
57. Mamidipudi, V., Cartwright, C.: A novel pro-apoptotic function of RACK1: suppression of Src activity in the intrinsic and Akt pathways. Oncogene **28**(50), 4421–4433 (2009)
58. Chang, B., Conroy, K., Machleder, E., Cartwright, C.: RACK1, a receptor for activated C kinase and a homolog of the beta subunit of G proteins, inhibits activity of src tyrosine kinases and growth of NIH 3T3 cells. Mol Cell Biol **18**(6), 3245–56 (1998)
59. Li, J., Xie, D.: RACK1, a Versatile Hub in Cancer. in press
60. Berns, H., Humar, R., Hengerer, B., Kiefer, F., Battegay, E.: RACK1 is up-regulated in angiogenesis and human carcinomas. FASEB J **14**(15), 2549–2558 (2000)
61. Mamidipudi, V., Dhillon, N., Parman, T., Miller, L., Lee, K., Cartwright, C.: RACK1 inhibits colonic cell

growth by regulating Src activity at cell cycle checkpoints. Oncogene **26**(20), 2914–2924 (2006)
62. Cao, X., Xu, J., Xu, J., Liu, X., Cheng, Y., Li, Q., Xu, Z., Liu, X.: RACK1 promotes breast carcinoma migration/metastasis via activation of the rhoa/rho kinase pathway. Breast Cancer Res Treat **126**(3), 555–563 (2010)
63. Shi, S., Deng, Y., Zhao, J., Ji, X., Shi, J., Feng, Y., Li, G., Li, J., Zhu, D., Koeffler, H., Zhao, Y., Xie, D.: RACK1 promotes non-small-cell lung cancer tumorigenicity through activating sonic hedgehog signaling pathway. J Biol Chem **287**(11), 7845–7858 (2012)
64. Wehner, P., Shnitsar, I., Urlaub, H., Borchers, A.: RACK1 is a novel interaction partner of PTK7 that is required for neural tube closure. Development **138**(7), 1321–1327 (2011)
65. Zhu, S.J., Pearson, B.J.: The Retinoblastoma pathway regulates stem cell proliferation in freshwater planarians. Dev Biol **373**(2), 442–452 (2013)
66. Wagner, D.E., Ho, J.J., Reddien, P.W.: Genetic regulators of a pluripotent adult stem cell system in planarians identified by RNAi and clonal analysis. Cell Stem Cell **10**(3), 299–311 (2012)
67. Reddien, P.W., Bermange, A.L., Murfitt, K.J., Jennings, J.R., Sánchez Alvarado, A.: Identification of genes needed for regeneration, stem cell function, and tissue homeostasis by systematic gene perturbation in planaria. Dev Cell **8**(5), 635–649 (2005)
68. Nicolas, E.: RbAp48 belongs to the histone deacetylase complex that associates with the retinoblastoma protein. J Biol Chem **275**(13), 9797–9804 (2000)
69. Hoek, M., Stillman, B.: Chromatin assembly factor 1 is essential and couples chromatin assembly to DNA replication in vivo. Proc Natl Acad Sci U S A **100**(21), 12183–12188 (2003)
70. Richly, H., Aloia, L., Di Croce, L.: Roles of the polycomb group proteins in stem cells and cancer. Cell Death and Disease **2**(9), 204 (2011)
71. Luo, M., Ling, T., Xie, W., Sun, H., Zhou, Y., Zhu, Q., Shen, M., Zong, L., Lyu, G., Zhao, Y., Ye, T., Gu, J., Tao, W., Lu, Z., Grummt, I.: NuRD blocks reprogramming of mouse somatic cells into pluripotent stem cells. Stem Cells **31**(7), 1278–1286 (2013)
72. Pacifico, F., Paolillo, M., Chiappetta, G., Crescenzi, E., Arena, S., Scaloni, A., Monaco, M., Vascotto, C., Tell, G., Formisano, S., Leonardi, A.: RbAp48 is a target of nuclear factor-κB activity in thyroid cancer. J Clin Endocrinol Metab **92**(4), 1458–1466 (2007)
73. Kong, L., Yu, X.P., Bai, X.H., Zhang, W.F., Zhang, Y., Zhao, W.M., Jia, J.H., Tang, W., Zhou, Y.B., Liu, C.J.: RbAp48 is a critical mediator controlling the transforming activity of human papillomavirus type 16 in cervical cancer. J Biol Chem **282**(36), 26381–26391 (2007)
74. Cao, F., Chen, Y., Cierpicki, T., Liu, Y., Basrur, V., Lei, M., Dou, Y.: An Ash2L/RbBP5 heterodimer stimulates the MLL1 methyltransferase activity through coordinated substrate interactions with the MLL1 SET domain. PLoS ONE **5**(11), 14102 (2010)
75. Odho, Z., Southall, S.M., Wilson, J.R.: Characterization of a novel WDR5-binding site that recruits RbBP5 through a conserved motif to enhance methylation of histone h3 lysine 4 by mixed lineage leukemia protein-1. J Biol Chem **285**(43), 32967–32976 (2010)
76. Pugh, D.J., Ab, E., Faro, A., Lutya, P.T., Hoffmann, E., Rees, D.J.: DWNN, a novel ubiquitin-like domain, implicates RBBP6 in mRNA processing and ubiquitin-like pathways. BMC Struct Biol **6**(1), 1 (2006)
77. Nakamura, Y., Tanaka, H., Arakawa, H., Yamaguchi, T., Shiraishi, K., Fukuda, S., Matsui, K., Takei, Y.: A ribonucleotide reductase gene involved in a p53-dependent cell-cycle checkpoint for DNA damage. Nature **404**(6773), 42–49 (2000)
78. p53R2-dependent pathway for DNA synthesis in a p53-regulated cell cycle checkpoint: Yamaguchi, t and matsuda, k and sagiya, y and iwadate, m and fujino, ma and nakamura, y and arakawa, h. Cancer Res **61**(22), 8256–62 (2001)
79. Guittet, O., Hakansson, P., Voevodskaya, N., Fridd, S., Graslund, A., Arakawa, H., Nakamura, Y., Thelander, L.: Mammalian p53R2 protein forms an active ribonucleotide reductase in vitro with the R1 protein, which is expressed both in resting cells in response to DNA damage and in proliferating cells. J Biol Chem **276**(44), 40647–40651 (2001)
80. Cho, E., Kuo, M., Liu, X., Yang, L., Hsieh, Y., Wang, J., Cheng, Y., Yen, Y.: Tumor suppressor FOXO3 regulates ribonucleotide reductase subunit RRM2B and impacts on survival of cancer patients. Oncotarget **5**(13), 4834–44 (2014)
81. Tian, H., Ge, C., Li, H., Zhao, F., Hou, H., Chen, T., Jiang, G., Xie, H., Cui, Y., Yao, M., Li, J.: Ribonucleotide reductase M2B inhibits cell migration and spreading by early growth response protein 1-mediated phosphatase and tensin homolog/Akt1 pathway in hepatocellular carcinoma. Hepatology **59**(4), 1459–1470 (2014)
82. Jørgensen, C., Ejlertsen, B., Bjerre, K., Balslev, E., Nielsen, D., Nielsen, K.: Gene aberrations of RRM1 and RRM2B and outcome of advanced breast cancer after treatment with docetaxel with or without gemcitabine. BMC Cancer **13**(1), 541 (2013)
83. Bourdon, A., Minai, L., Serre, V., Jais, J., Sarzi, E., Aubert, S., Chrétien, D., de Lonlay, P., Paquis-Flucklinger, V., Arakawa, H., Nakamura, Y., Munnich, A., Rötig, A.: Mutation of RRM2B, encoding p53-controlled ribonucleotide reductase (p53R2), causes severe mitochondrial DNA depletion. Nat Genet **39**(6), 776–780 (2007)
84. Shaibani, A., Shchelochkov, O., Zhang, S., Katsonis, P., Lichtarge, O., Wong, L., Shinawi, M.: Mitochondrial neurogastrointestinal encephalopathy due to mutations in RRM2B. Arch Neurol **66**(8), 1028–32 (2009)
85. Bornstein, B., Area, E., Flanigan, K., Ganesh, J., Jayakar, P., Swoboda, K., Coku, J., Naini, A., Shanske, S., Tanji, K., Hirano, M., DiMauro, S.: Mitochondrial DNA depletion syndrome due to mutations in the RRM2B gene. Neuromuscul Disord **18**(6), 453–459 (2008)
86. Finsterer, J., Ahting, U.: Mitochondrial depletion syndromes in children and adults. Can J Neurol Sci **40**(5), 635–44 (2013)

87. Inuzuka, M., Hayakawa, M., Ingi, T.: Serinc, an activity-regulated protein family, incorporates serine into membrane lipid synthesis. J Biol Chem **280**(42), 35776–35783 (2005)
88. Rossman, T., Wang, Z.: Expression cloning for arsenite-resistance resulted in isolation of tumor-suppressor fau cdna: possible involvement of the ubiquitin system in arsenic carcinogenesis. Carcinogenesis **20**(2), 311–316 (1999)
89. Andreu-Agullo, C., Maurin, T., Thompson, C., Lai, E.: Ars2 maintains neural stem-cell identity through direct transcriptional activation of Sox2. Nature **481**(7380), 195–198 (2011)
90. Wilson, M., Wang, D., Wagner, R., Breyssens, H., Gertsenstein, M., Lobe, C., Lu, X., Nagy, A., Burke, R., Koop, B., Howard, P.: ARS2 is a conserved eukaryotic gene essential for early mammalian development. Mol Cell Biol **28**(5), 1503–1514 (2008)
91. Chi, B., Wang, Q., Wu, G., Tan, M., Wang, L., Shi, M., Chang, X., Cheng, H.: Aly and THO are required for assembly of the human TREX complex and association of TREX components with the spliced mRNA. Nucleic Acids Res **41**(2), 1294–1306 (2012)
92. Jimeno, S., Aguilera, A.: The THO complex as a key mRNP biogenesis factor in development and cell differentiation. J Biol **9**(1), 6 (2010)
93. Wang, L., Miao, Y., Zheng, X., Lackford, B., Zhou, B., Han, L., Yao, C., Ward, J., Burkholder, A., Lipchina, I., Fargo, D., Hochedlinger, K., Shi, Y., Williams, C., Hu, G.: The THO complex regulates pluripotency gene mRNA export and controls embryonic stem cell self-renewal and somatic cell reprogramming. Cell Stem Cell **13**(6), 676–690 (2013)
94. Domínguez-Sánchez, M., Sáez, C., Japón, M., Aguilera, A., Luna, R.: Differential expression of THOC1 and ALY mRNP biogenesis/export factors in human cancers. BMC Cancer **11**(1), 77 (2011)
95. Allton, K., Jain, A.K., Herz, H.M., Tsai, W.W., Jung, S.Y., Qin, J., Bergmann, A., Johnson, R.L., Barton, M.C.: Trim24 targets endogenous p53 for degradation. Proc Natl Acad Sci U S A **106**(28), 11612–11616 (2009)
96. Torres-Padilla, M.E., Zernicka-Goetz, M.: Role of TIF1 as a modulator of embryonic transcription in the mouse zygote. J Cell Biol **174**(3), 329–338 (2006)
97. Khetchoumian, K., Teletin, M., Tisserand, J., Mark, M., Herquel, B., Ignat, M., Zucman-Rossi, J., Cammas, F., Lerouge, T., Thibault, C., Metzger, D., Chambon, P., Losson, R.: Loss of trim24 (tif1α) gene function confers oncogenic activity to retinoic acid receptor alpha. Nat Genet **39**(12), 1500–1506 (2007)
98. Tsai, W.W., Wang, Z., Yiu, T.T., Akdemir, K.C., Xia, W., Winter, S., Tsai, C.Y., Shi, X., Schwarzer, D., Plunkett, W., Aronow, B., Gozani, O., Fischle, W., Hung, M., Patel, D.J., Barton, M.C.: TRIM24 links a non-canonical histone signature to breast cancer. Nature **468**(7326), 927–932 (2010)
99. Kikuchi, M., Okumura, F., Tsukiyama, T., Watanabe, M., Miyajima, N., Tanaka, J., Imamura, M., Hatakeyama, S.: TRIM24 mediates ligand-dependent activation of androgen receptor and is repressed by a bromodomain-containing protein, BRD7, in prostate cancer cells. Biochim Biophys Acta **1793**(12), 1828–1836 (2009)
100. Xie, P.: TRAF molecules in cell signaling and in human diseases. J Mol Signal **8**(1), 7 (2013)
101. Li, L., Liao, J., Ruland, J., Mak, T.W., Cohen, S.N.: A TSG101/MDM2 regulatory loop modulates MDM2 degradation and MDM2/p53 feedback control. Proc Natl Acad Sci U S A **98**(4), 1619–1624 (2001)
102. Cheng, X.: Role of TSG101 in cancer. Front Biosci **18**(1), 279 (2013)
103. Hu, R.J., Lee, M.P., Connors, T.D., Johnson, L.A., Burn, T.C., Su, K., Landes, G.M., Feinberg, A.P.: A 2.5-Mb transcript map of a tumor-suppressing subchromosomal transferable fragment from 11p15.5, and isolation and sequence analysis of three novel genes. Genomics **46**(1), 9–17 (1997)
104. Wang, D.C., Wang, H.F., Yuan, Z.N.: Runx2 induces bone osteolysis by transcriptional suppression of TSSC1. Biochem Biophys Res Commun **438**(4), 635–639 (2013)
105. Zhou, H., Clapham, D.E.: Mammalian MagT1 and TUSC3 are required for cellular magnesium uptake and vertebrate embryonic development. Proc Natl Acad Sci U S A **106**(37), 15750–15755 (2009)
106. Horak, P., Tomasich, E., Vaňhara, P., Kratochvílová, K., Anees, M., Marhold, M., Lemberger, C.E., Gerschpacher, M., Horvat, R., Sibilia, M., Pils, D., Krainer, M.: TUSC3 loss alters the ER stress response and accelerates prostate cancer growth *in vivo*. Sci Rep **4**(3739) (2014)
107. Vaňhara, P., Horak, P., Pils, D., Anees, M., Petz, M., Gregor, W., Zeillinger, R., Krainer, M.: Loss of the oligosaccharyl transferase subunit TUSC3 promotes proliferation and migration of ovarian cancer cells. Int J Oncol **42**(4), 1383–1389 (2013)
108. Loddo, S., Parisi, V., Doccini, V., Filippi, T., Bernardini, L., Brovedani, P., Ricci, F., Novelli, A., Battaglia, A.: Homozygous deletion in TUSC3 causing syndromic intellectual disability: a new patient. Am J Med Genet **161**(8), 2084–2087 (2013)
