# Supplementary material for: Digital gene expression approach over multiple RNA-Seq data sets to detect neoblast transcriptional changes in Schmidtea mediterranea
Source: BMC Genomics. 2015 May 8;16(1):361. doi: 10.1186/s12864-015-1533-1 (PMC4494696; doi:10.1186/s12864-015-1533-1)

Smed454 DGE Expression Levels [X1 vs X2 vs Xin]

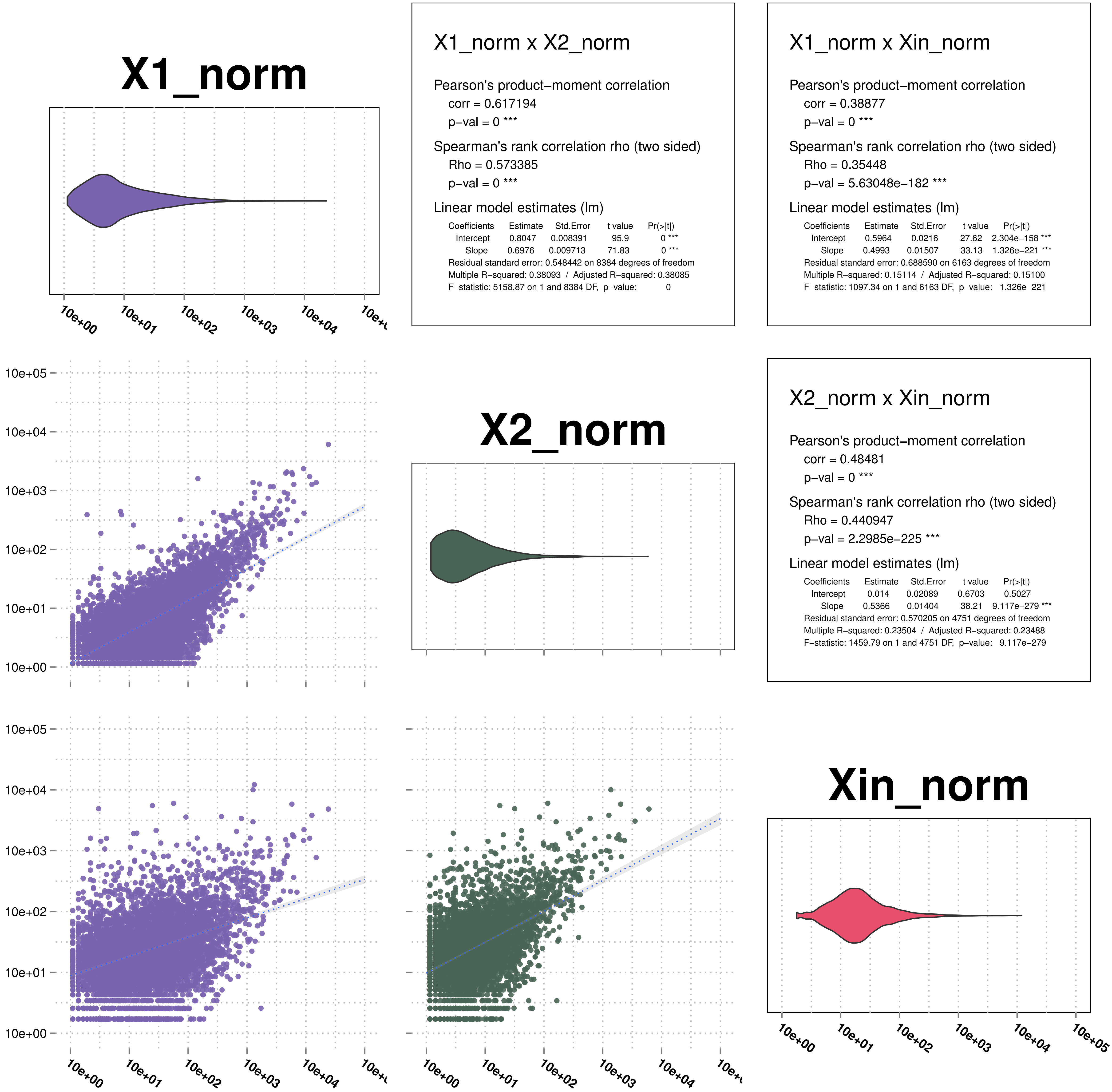

Supplement: Additional file 14 — Pearson and Spearman correlations of the normalized expression levels among X1, X2 and Xin. Diagonal panels show violin plots with the distribution of the normalized expression levels for each of the three cell populations data sets. Panels on the upper diagonal summarize both Pearson (parametric) and Spearman (non-parametric) correlations, along with the p-values and the linear regression model estimates for the pairwise comparison between data sets. On the bottom diagonal panels, for each pair of cell fractions the scatterplots show differences in expression for each DGE tag. Blue dotted line is defined by the intercept and slope values for the linear regression model presented on the corresponding upper panel, confidence interval is drawn as a grey shadow along that regression line. Those tags having a normalized expression value of zero in one or both of the cell types, when considering each pair-wise comparisons, were removed before computing correlations and for the plots. One can notice that X1 and X2 are the more correlated pair of cell fractions, then X2 and Xin, and finally X1 and Xin. Those results match to what would be expected. [file 12864_2015_1533_MOESM14_ESM.pdf]
