# Supplementary material for: Digital gene expression approach over multiple RNA-Seq data sets to detect neoblast transcriptional changes in Schmidtea mediterranea
Source: BMC Genomics. 2015 May 8;16(1):361. doi: 10.1186/s12864-015-1533-1 (PMC4494696; doi:10.1186/s12864-015-1533-1)
Supplement: Additional file 13 — Splashplot projection of the X1/X2 versus Xin expression changes of upregulated contigs by cell population. A - Splashplot for overrepresented contigs in the three cell fractions X1, X2 and Xin according to our DGE data over the Smed454 transcriptome [10]. B - Same representation using the data published by Labbé [14]. Both plots show a similar composition, revealing a low number of transcripts overexpressed specifically in X2/progeny cells. [file 12864_2015_1533_MOESM13_ESM.pdf]

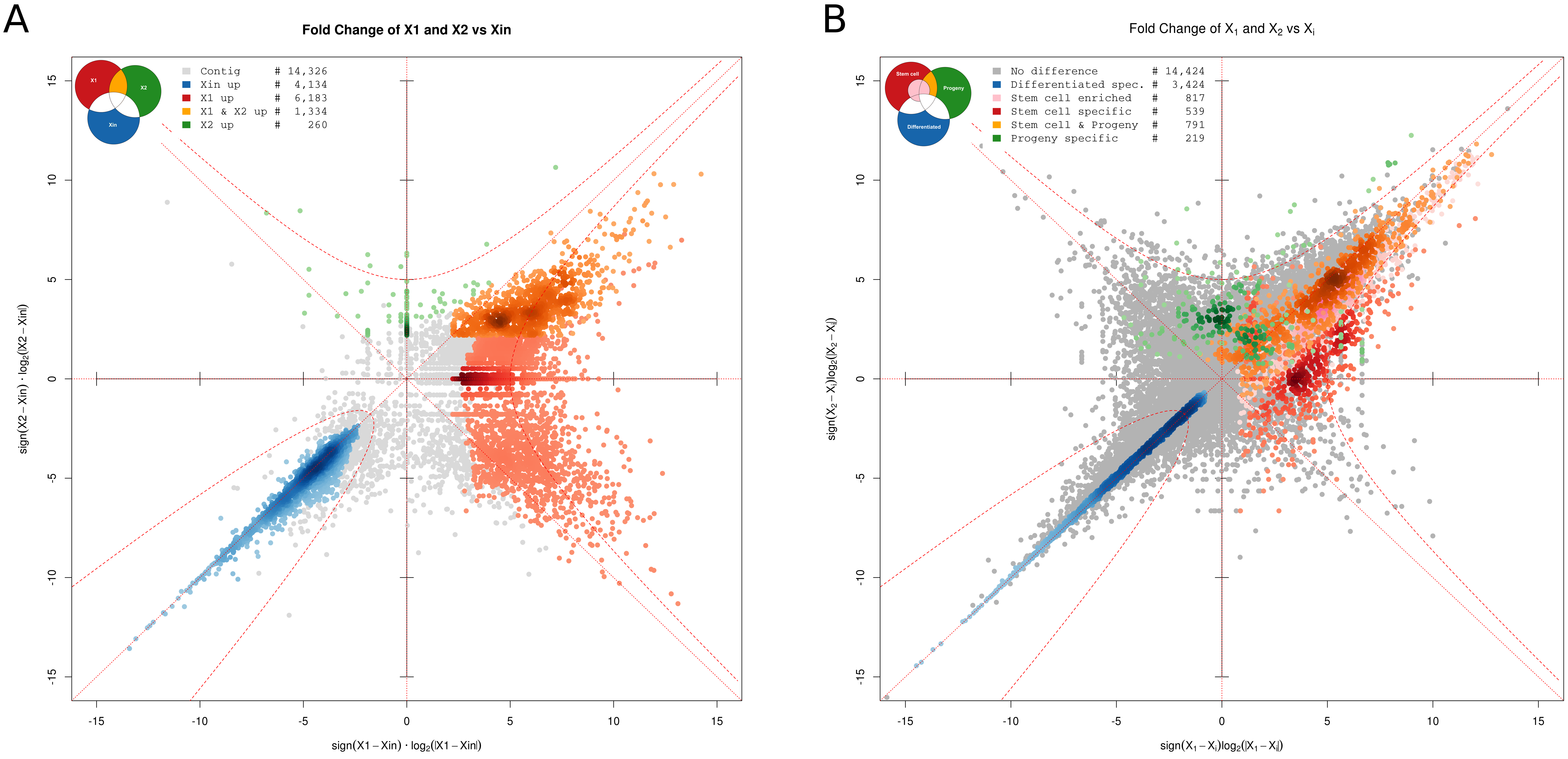

A
Fold Change of X1 and X2 vs Xin
X1
X2
Xin
Contig # 14,326
Xin up # 4,134
X1 up # 6,183
X1 & X2 up # 1,334
X2 up # 260
sign(X2 – Xin) · log2(|X2 – Xin|)
sign(X1 – Xin) · log2(|X1 – Xin|)
B
Fold Change of X1 and X2 vs Xi
Stem cell
Progeny
Differentiated
No difference # 14,424
Differentiated spec. # 3,424
Stem cell enriched # 817
Stem cell specific # 539
Stem cell & Progeny # 791
Progeny specific # 219
sign(X2 – Xi)log2(|X2 – Xi|)
sign(X1 – Xi)log2(|X1 – Xi|)
